# Cancer-associated fibroblast subtypes differentially modulate natural killer cells in cancer

**DOI:** 10.1016/j.celrep.2026.117527

**Published:** 2026-06-11

**Authors:** Leonor Nunes Rodrigues, Hafsa Munir, Ibone Thate Arrazola, Matthias M. Gaida, Christoph Eckert, Jerome Boulanger, Ana C.F. Ferreira, Andrew N.J. McKenzie, Jacqueline D. Shields

**Affiliations:** 1 MRC Laboratory of Molecular Biology, Cambridge CB2 0QH, UK; 2 MRC Cancer Unit, Hutchison/MRC Research Centre, University of Cambridge, Cambridge, UK; 3 Helmholtz Institute for Translational Oncology Mainz (HI-TRON Mainz) – a Helmholtz Institute of the DKFZ, Mainz, Germany; 4 German Cancer Research Centre (DKFZ), Division of Dermal Oncoimmunology, Heidelberg, Germany; 5 Institute of Pathology, University Medical Center Mainz, JGU-Mainz 55131 Mainz, Germany; 6 TRON, Translational Oncology at the University Medical Center, JGU-Mainz 55131 Mainz, Germany; 7 Research Center for Immunotherapy, University Medical Center Mainz, JGU-Mainz 55131 Mainz, Germany; 8 Comprehensive Cancer Centre, Kings College London, London, UK; 9 Centre for Cancer Sciences, School of Medicine, Biodiscovery Institute, University of Nottingham, Nottingham, UK

## Abstract

Natural killer (NK) cells are cytotoxic innate lymphoid cells which directly kill tumor cells, thus represent an attractive target for immunotherapy. However, NK cells face immunosuppression in the tumor microenvironment (TME), rendering them dysfunctional. While cancer-associated fibroblasts (CAFs) represent an abundant, heterogeneous component of pancreatic ductal adenocarcinoma (PDAC), their interplay with NK cells is largely understudied. Analyzing human samples and employing mouse models of PDAC and functional assays, we observed that intratumoral NK cells are immature, and TGF-**β** driven myofibroblastic (my)CAFs are strong NK suppressors, in contrast to inflammatory (i)CAF. Furthermore, myCAF-enriched tumor areas excluded NK cells, consistent with their reduced capacity to attract NK cells. Pancreatic CAFs in general reduced NK cell cytotoxicity by direct contact and via soluble factors, including prostaglandin E2 (PGE2). This work reveals distinct and overlapping roles of CAF subpopulations on NK cell functions, suggesting that overcoming CAF-imposed barriers to NK cytotoxicity and tumor infiltration is essential to unleash their anti-tumoral properties.

## Graphical abstract

**Graphical abstract:**
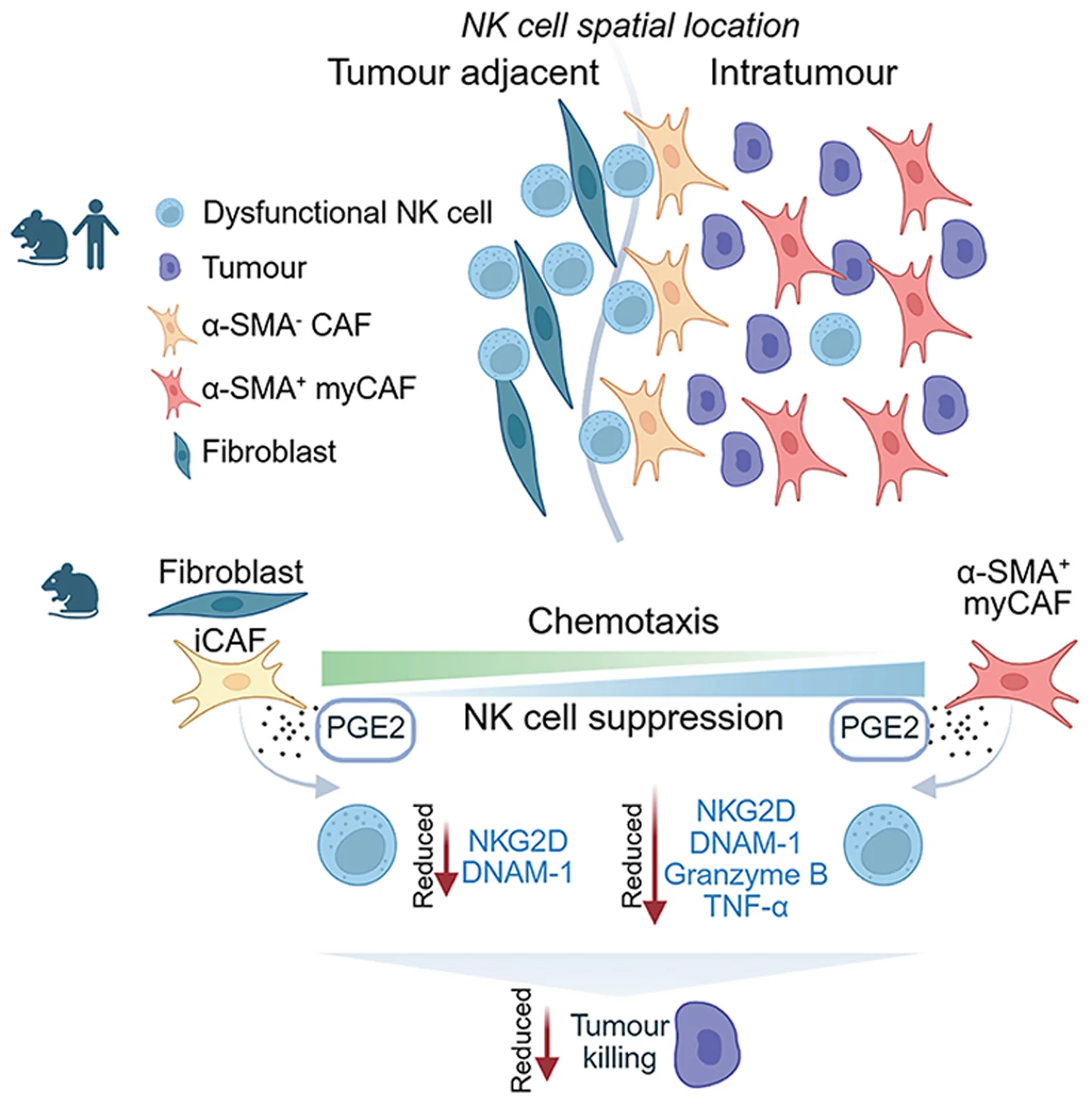

## Introduction

Immunotherapy has revolutionized treatment of cancers such as melanoma,^1,2^ but their impact on solid cancers including pancreatic ductal adenocarcinoma (PDAC) has been limited.^2^ PDAC remains a deadly disease with a 5-year survival rate of only 13%.^3^

One of the main therapeutic challenges in PDAC is the suppressive tumor microenvironment (TME).^4^ This includes cancer associated fibroblasts (CAFs)^5^ which constitute a major component of PDAC and promote tumor growth, immune cell infiltration, and suppression, while also impairing drug penetration due to extracellular matrix (ECM) deposition and modulation, known as desmoplasia.^6^ Therefore, targeting CAFs is an attractive approach to reprogram the TME, restoring infiltration and anti-tumor functions of infiltrating immune cells.^7,8^

CAFs are highly heterogeneous across several cancer types and lack specific single phenotypic markers.^9–11^ Two functionally distinct CAF subtypes were first identified in PDAC: tumor adjacent myofibroblastic CAFs (myCAFs) and inflammatory CAFs (iCAFs) at the margins of tumors.^12^ myCAF and iCAF are shared between solid cancers and are induced *in vitro* with transforming growth factor β (TGF-β) and interleukin (IL)-1α/tumour necrosis factor (TNF)-α, respectively.^12,13^ While myCAF contribute to ECM deposition, iCAF release immunosuppressive factors.^12,13^ myCAFs are identified by α-smooth muscle actin (α-SMA), while iCAFs express lower levels of α-SMA,^12^ express CD34,^10^ Ly6C,^13^ high levels of platelet-derived growth factor receptor-α (PDGFR-α),^11^ IL-6, and leukemia inhibitory factor (LIF).^13^ Antigen-presenting CAFs (apCAFs) express major histo-compatibility class-II (MHC-II) and present antigens to T cells, inducing Treg expansion.^11,14^

Natural killer (NK) cells belong to the innate lymphoid cell (ILC) family and are important for controlling viral infections and cancer incidence.^15–17^ Unlike T cells, NK cells show a variety of germline-encoded activator and inhibitory receptors which enable them to recognize other cells without antigen recognition.^18^ Although, cancer cells often upregulate stress ligands and downmodulate MHC-I to avoid T cell-mediated immune surveillance,^19^ this makes them susceptible to NK cell lysis by engagement of activating receptors such as NKG2D and DNAM-1 with their respective cancer cell-expressed ligands.^19^ NK cells also recruit type 1 conventional dendritic cells (cDC1s) intratumorally to promote adaptive immune responses.^20^ Notably, in contrast to T cells, NK cells do not need to be human leukocyte antigen (HLA) haplotype matched between donor and patient, offering the possibility of “off-the-shelf” therapies.^21^ While adoptive transfer of NK cells into patients with hematological cancers showed some success,^22^ solid tumors remain a challenge due to poor NK infiltration and TME suppression.^23–25^

The roles of NK cells in PDAC remain poorly understood.^26^ Despite their low proportion intratumorally, a type I and II IFN signature in NK cells correlates with survival,^27^ while NK cell infiltration in pancreatic tumor models via CXCR2 overexpression reduces tumor growth.^28,29^ Previous studies suggest that CAFs suppress NK cell anti-tumor functions, but little is known about where NK cells localize in PDAC, and whether distinct CAF subtypes contribute to their functional dysregulation. For example, CAFs suppress NK cells by downmodulating DNAM-1 ligand (CD155) on their surface by direct contact,^30^ or via prostaglandin E2 (PGE2) and indoleamine 2,3-dioxygenase (IDO),^31^ while CAF-derived TGF-β affects several NK cell functions.^32^ Indirectly, CAFs were shown to promote type 2 macrophage polarization which inhibits NK cells.^33^ One study showed that netrin G1 in CAFs rescues NK cell anti-tumor functions by restoring CAF-derived IL-15 secretion,^34^ illustrating the importance of the CAF-NK cell axis.

Here, we investigated how pancreatic CAFs and distinct subpopulations modulate anti-tumor functions of NK cells in PDAC, analyzing human samples, employing syngeneic and autochthonous mouse models and functional assays. We show that CAFs, particularly myCAF, modulate NK cell phenotype and killing capacity which is partially mediated by PGE2.

## Results

### NK cells are excluded from intratumoral regions in human PDAC

**Figure 1:**
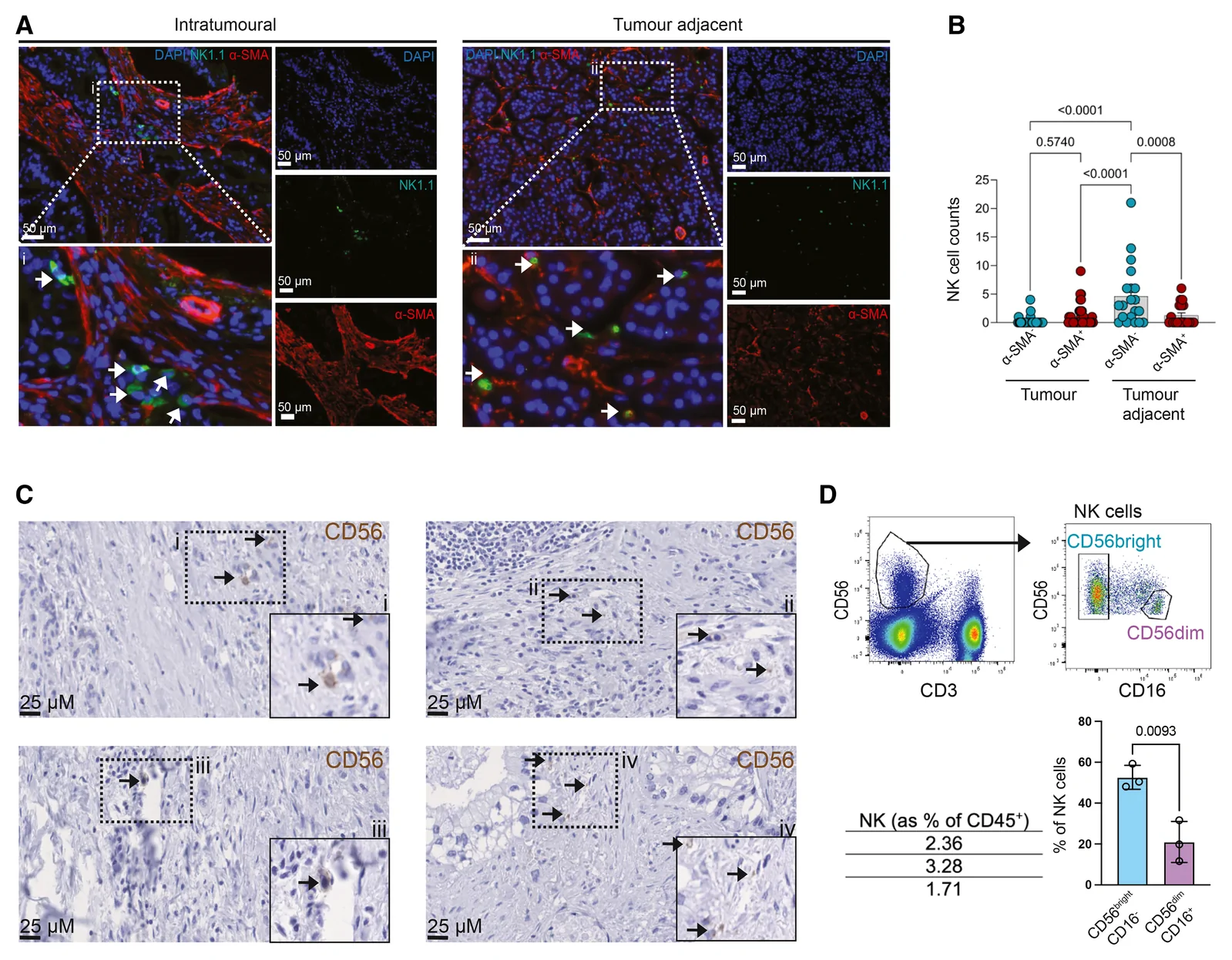
NK cells are rare infiltrates in human PDAC showing a non-cytotoxic profile (A) Immunofluorescence staining of human TMA containing 34 PDAC and 27 cancer-adjacent pancreatic tissues. Example where NK cells are identified in tumor and tumor-adjacent cores near identified with α-SMA marker; scale bars, 50 μM. (B) Quantification of NK cells in the TMA described in (A). (C) Immunohistochemical staining with CD56 (brown, indicated by arrows) of NK cells in human PDAC TMA counterstained with hematoxylin (*n* = 10 tumor core cases). Zoomed areas of interest on bottom right of images; scale bars, 25 μM. (D) Flow cytometry quantification of NK cell CD56^dim^ vs. CD56^bright^ populations in human PDAC samples, *n* = 3 and percentage of NK cells (of CD45^+^) in tumors analyzed, *n* = 3. Data shown as mean ± SEM and statistical significance determined with one-way ANOVA with Tukey’s multiple comparison or two-tailed unpaired *t* test for *p* values ≤0.05. Relevant statistics shown.

To interrogate NK cell infiltration in PDAC, we analyzed human tissue microarrays (TMAs) containing 34 PDAC and 27 cancer-adjacent pancreatic tissue by immunofluorescence (IF) (Figure 1A). Very few NK cells were present and accumulated mostly in tumor-adjacent tissue rather than tumor cores, suggesting tumor exclusion (Figure 1B). Using α-SMA staining and morphologically excluding pericytes (Figure S1A), we verified that NK cells did not associate with α-SMA-rich areas (Figures 1A and 1B). An additional PDAC TMA also revealed very few NK cells by H&E and CD56 staining in tumor cores (Figure 1C), and whole section imaging of PDAC tissue samples followed by H&E and CD56 staining revealed that the few NK cells identified in tumors were located predominantly at the invasive margins of tumors, embedded in stroma (Figure S1B). Analysis of these tumors by flow cytometry revealed that CD56^+^ CD3^–^ NK cells^15^ constitute only 2%–3% of CD45^+^ immune cells (Figures 1D; Figure S1C). Interestingly, tumors show an accumulation of 40%–60% CD56^bright^ CD16^−^ NK cells (Figure 1D), which are reported to have lower cytotoxic capacity than blood-circulating CD56^dim^CD16^+^ NK cells.^35^ Overall, NK cells are rare infiltrates in human PDAC, containing immature subpopulations and primarily locating in stromal regions at tumor invasive margins with no direct association with α-SMA^+^ CAFs.

### NK cells show an immature phenotype while locating distant to α-SMA^+^ CAF regions in murine tumor models

**Figure 2:**
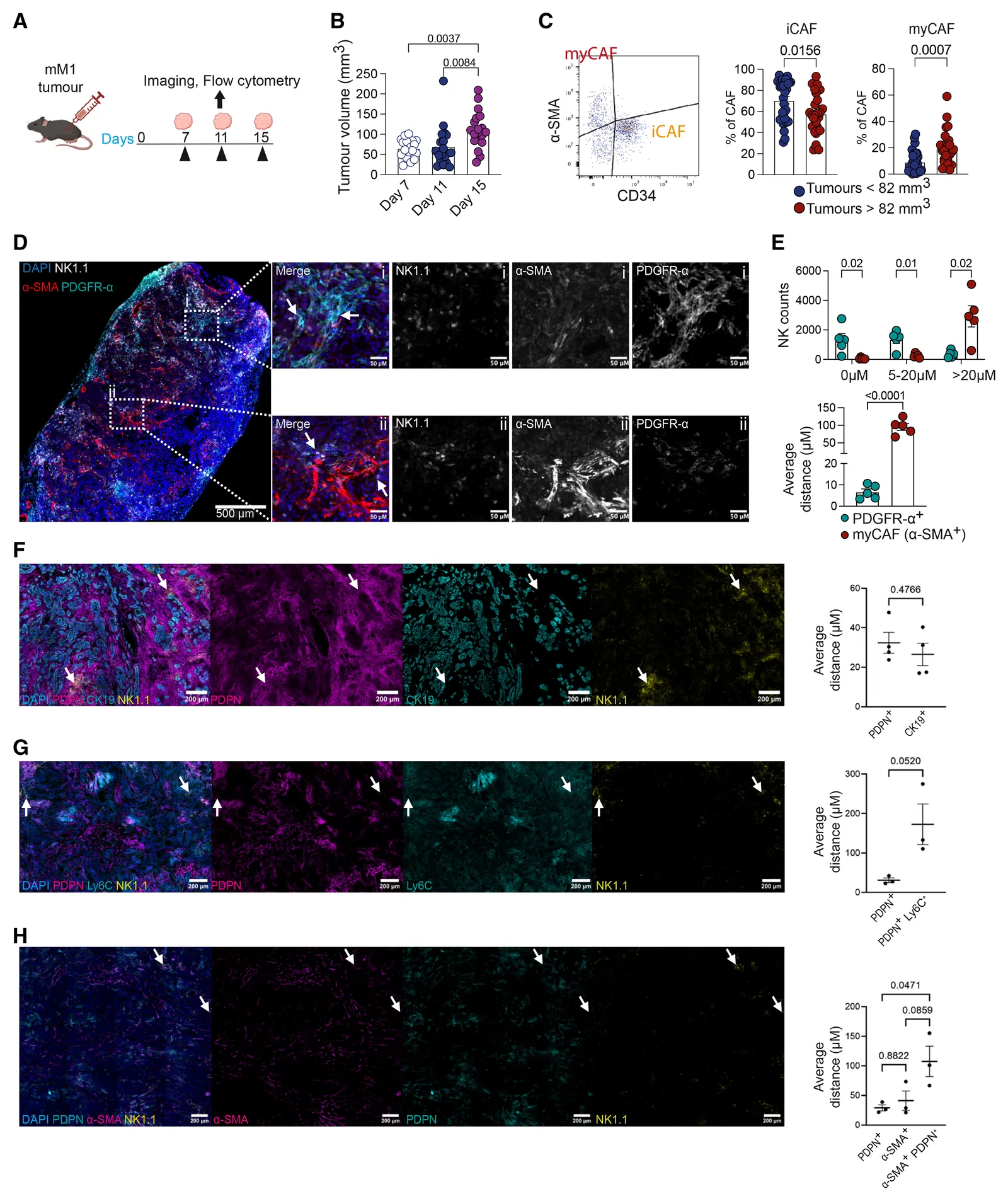
NK cells locate near PDPN^+^/PDGFR-α^+^ CAF in murine pancreatic cancer models (A) Illustration of syngeneic tumor model (created with Biorender.com). (B) Tumor volumes (mm^3^) at different time points. 6 independent experiments pooled; *n* = 18, *n* = 19 and *n* = 19 for days 7, 11, and 13–15, respectively. (C) Quantification of CAF subtypes based on α-SMA and CD34 (gating on Figures S2A and S2B); stratification per average tumor volume (82 mm^3^). (D and E) Imaging of mM1 tumors comparing NK cell position relative to CAF, 20× magnification. DAPI (blue), PDGFR-α (cyan), α-SMA (red), and NK1.1 (white), scale bars, 500 μM. Individual channels for respective regions of interest in grayscale, scale bars, 50 μM. (E) NK cell counts at specific distances to each CAF subtype and average distance of each NK cell to the closest CAF region, *n* = 5 tumors. (F–H) Examples of IF imaging of autochthonous KPC tumors, 20× magnification, illustrating NK cell position relative to CAF markers and CK19; scale bars, 200 μM, arrows indicate NK cells. Individual channel panels shown as indicated. Quantification of average distance of NK cells to the respective marker or combination on the right of each imaging panel. (F) DAPI (blue), PDPN (magenta), CK19 (cyan), and NK1.1 (yellow), *n* = 4 tumors analyzed. (G) DAPI (blue), PDPN (magenta), Ly6C (cyan), and NK1.1 (yellow), *n* = 3. (H) DAPI (blue), α-SMA (magenta), PDPN (cyan), and NK1.1 (yellow), *n* = 3. Data shown as mean ± SEM and statistical significance determined with one-way ANOVA with Tukey’s multiple comparison or two-tailed unpaired *t* test for *p* values ≤0.05. Relevant statistics shown.

Due to limited number of patient samples, we profiled NK cells and CAFs in murine tumors to provide mechanistic insight into their potential crosstalk. We utilized a subcutaneous syngeneic model, implanting LSL-Kras^G12D/+^;LSLTp53^R172H/+^;Pdx-1-Cre (KPC)-derived tumor cells from a metastatic tumor (the mM1 cell line).^36^ Although there are limitations, these subcutaneously implanted tumors are easier to manipulate than orthotopic tumors while retaining features of autochthonous parental KPC tumors, including a CAF-rich compartment/ECM deposition and similar immune cell composition/exclusion, due intrinsic immunosuppressive properties of KPC-derived tumors.^37^ We interrogated CAF heterogeneity at time points in subcutaneously implanted mM1 tumors (Figure 2A). We observed increased tumor volumes from day 7–15 (Figure 2B) and were able to detect CAFs by exclusion of immune CD45^+^ and CD31^+^ endothelial cells and by expression of podoplanin (PDPN) as a general CAF marker^11,13^ and α-SMA as a marker of myofibroblastic (my)-CAF^12^ (Figure 2C). CD34 and α-SMA expression were then used to distinguish inflammatory (i)CAFs (CD34^+^ α-SMA^–/low^) from my-CAFs (CD34^–^ α-SMA^+^) (Figure 2C), as described before.^12,13^ Stratifying tumors by average volume indicated that tumors below average volume contained higher frequencies of iCAFs, whereas larger tumors harbored significantly more myCAFs (Figure 2C). This was confirmed using Ly6C as an alternative iCAF marker (Figures S2A–S2D). Moreover, apCAF marker MHC-II^11^ was also detected in this model, supporting retention of heterogeneity (Figure S2D). Imaging of tumors revealed that PDGFR-α^+^ α-SMA^–/low^ CAFs were most abundant, while spindle-shaped PDGFR-α^–/low^ α-SMA^+^ cells (corresponding to myCAFs) were fewer and mostly detected in intratumoral areas (Figures S2E and S2F). These tumors also retained ECM deposition in stromal areas, as indicated by perlecan staining (Figure S2F), corroborating the presence of stromal content, as reported before.^37^

We assessed NK cell colocalization with CAFs in both syngeneic mM1 (Figures 2D and 2E) and autochthonous KPC models (Figures 2F–2H). In whole sections of mM1 tumors, NK cells associated with PDGFR-α^+^ α-SMA^–/low^ CAFs (Figures 2D and 2E). In contrast, NK cells were not frequently detected near α-SMA^+^ myCAFs (Figures 2D and 2E). In the pancreas, intratumoral regions contained fewer NK cells which were at an average distance of 20–30 μM to PDPN, delineating CAFs in general, and cytokeratin 19 (CK19), identifying ductal epithelial cancer cells (Figure 2F). Despite proximity with general CAF marker PDPN, NK cells did not associate with specific CAF subtypes PDPN^+^Ly6C^+^ (iCAF) or PDPN^+^α-SMA^+^ (myCAF) in the autochthonous model (Figures 2G and 2H). This corroborates observations in human tumors where NK cells were identified in stromal areas near tumors (Figure S2B) but detected in lower frequency in dense intratumoral areas delineated by α-SMA, compared to less dense cancer-adjacent tissue (Figure 1B).

**Figure 3:**
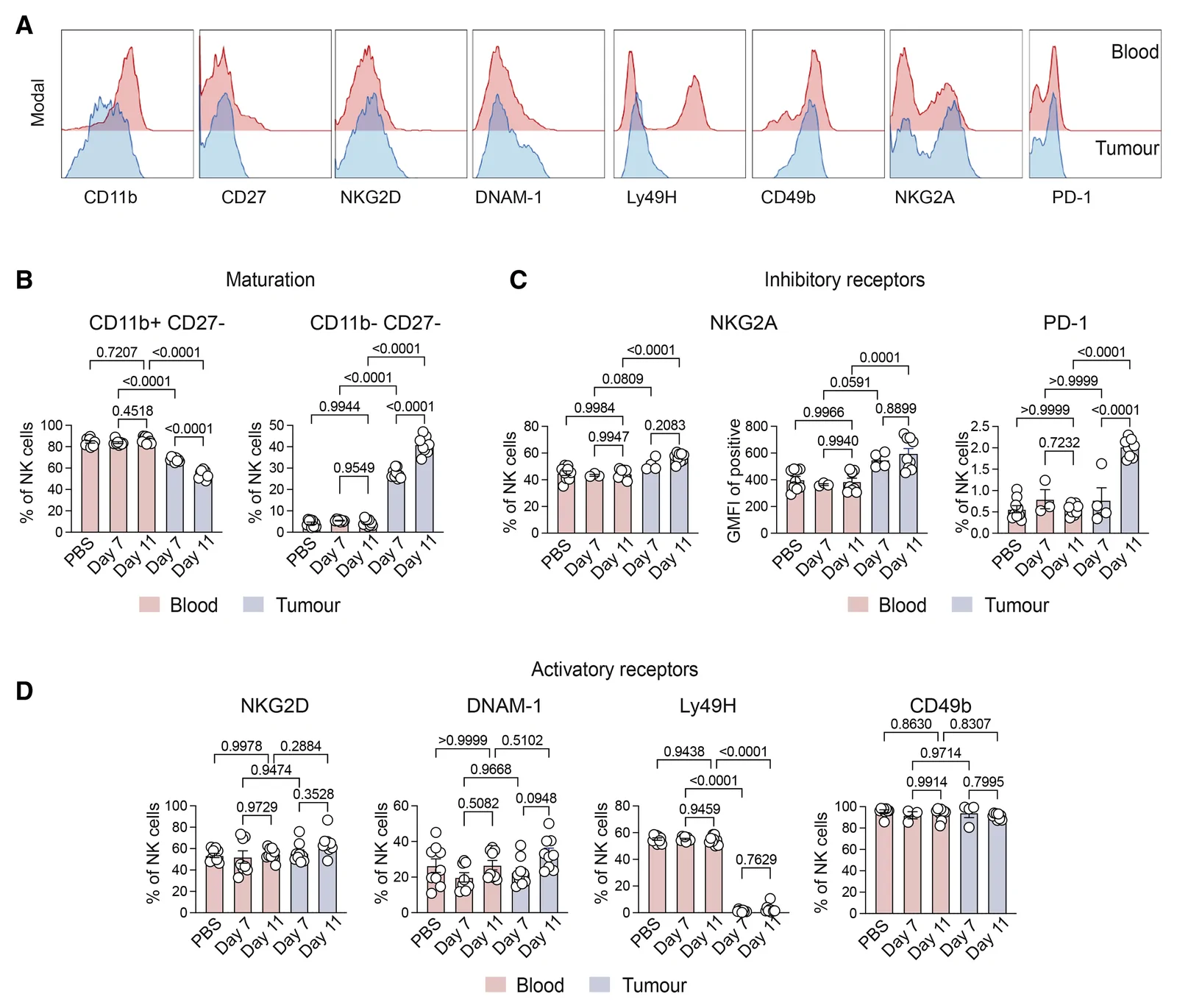
Murine NK cells show an immature non-cytotoxic profile in mM1 syngeneic tumors (A) Flow cytometry histograms of murine NK cell markers comparing blood with tumor (gating and controls on Figures S3A–S3C). (B-D) NK cell profile comparing days 7 and 11 tumors with blood counterparts and blood of non-tumor bearing mice: (B) maturation markers as frequency of CD11b^+^ CD27^−^ mature NK cells and CD11b^−^ CD27^−^ immature NK cells; (C) frequency and GMFI of NKG2A^+^ and frequency of PD-1^+^ NK cells; (D) frequency of activation markers NKG2D, DNAM-1, Ly49H, and CD49b. (B–D) 2 independent experiments pooled, *n* = 9 for all except blood day 7 (*n* = 8; one sample excluded due to insufficient material). (C and D) Data from 1 experiment for day 7 PD-1 and CD49b (*n* = 4 tumor; *n* = 3 blood: insufficient material for analysis); 2 independent experiments for day 11 (*n* = 9). All data shown as mean ± SEM and statistical significance determined with one-way ANOVA with Tukey’s multiple comparison or two-tailed unpaired *t* test for *p* values ≤0.05. Relevant statistics shown.

Next, we determined if the TME could influence NK cell profile by comparing tumor with blood NK cells in the mM1 model (Figures 3A–3D). We selected NK cell markers known to be indicators of anti-tumor functions: mature CD11b^+^ CD27^–^ cytotoxic and immature non-cytotoxic CD11b^–^ CD27^–^ NK cells,^38^ corresponding to human CD56^dim^ and CD56^bright^, respectively^35^; markers of NK cell inhibition (e.g., NKG2A and PD-1) and activation (e.g., NKG2D, DNAM-1, Ly49H, and CD49b).^18^ NK cells were identified as CD3^−^ NK1.1^+^ or NKp46^15^ (Figure S3A). Day 7 tumors predominantly contained CD11b^+^ CD27^–^ mature NK cells, but by day 11 immature CD11b^–^ CD27^–^ NK cells dominated (Figures 3A and 3B; Figures S3B–S3D). Moreover, intratumoral NK cells showed a distinct functional profile when compared to circulating NK cells from tumor-bearing mice which maintain a CD11b^+^ CD27^–^ mature phenotype mirroring those in the blood of control mice (Figures 3A and 3B). A slightly higher frequency and expression of NKG2A was detected on tumor infiltrated NK cells compared with blood counterparts; PD-1, which was largely undetectable, was upregulated on about 2% of NK cells in day 11 tumors (Figure 3C). The frequency of NK cells expressing activating receptors NKG2D and DNAM-1 was comparable along tumor progression and within blood (Figure 3D). Interestingly, Ly49H was not detectable on intratumoral NK cells, in contrast to blood (Figure 3D). This could indicate either specific recruitment of Ly49H^−^ NK cell subsets, rapid downmodulation of Ly49H, or preferential proliferation of Ly49H^−^ NK cells intratumorally. Following reports of intratumoral NK cells converting into ILC1 via downregulation of CD49b,^39^ we also analyzed its expression, but found that intratumoral NK1.1^+^ NK cells retain CD49b in this model, similar to blood counterparts (Figure 3D). Together, these results suggest that NK cells, upon entering a tumor, are converted to an immature non-cytotoxic status, being identified in stromal regions but excluded from iCAF and myCAF rich areas.

### Pancreatic CAFs reduce NK cell cytotoxicity *in vitro*

**Figure 4:**
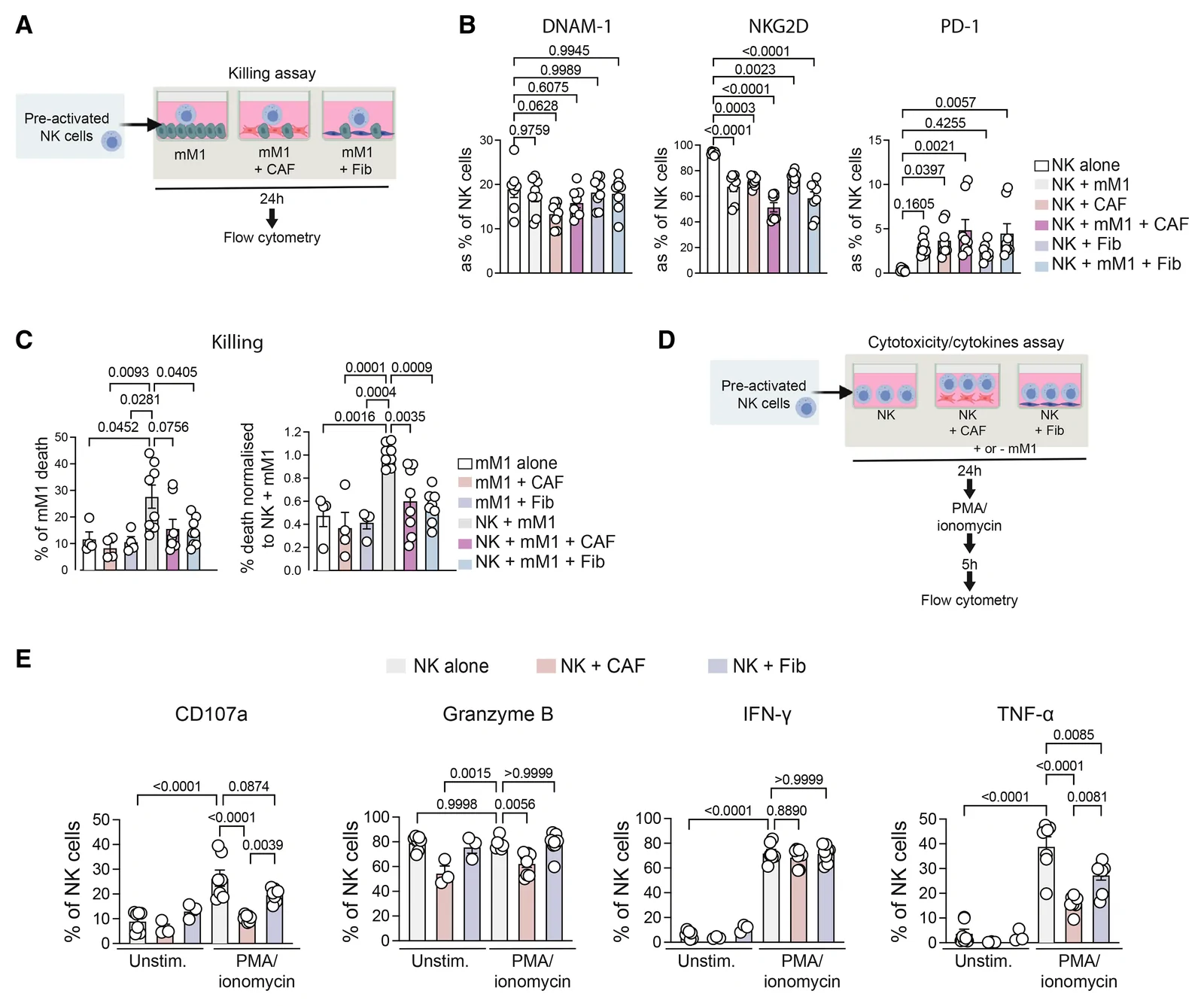
NK cell cytotoxic functions are suppressed by pancreatic CAFs (A)Illustration of method for tri-culture killing assays (created with Biorender.com). (B) NK cell receptor profile in killing assays: NKG2D, DNAM-1, and PD-1 as percentage of NK cells. n = 4 independent experiments, with technical duplicates, except for NK alone in one experiment. (C)Quantification of mM1 death in killing assay and normalized to “NK + mM1” for each experiment. n = 4 independent experiments; technical duplicates for conditions with NK cells. (D) Illustration of method for NK cell degranulation/cytokine release assay after 24 h of contact with Fib or CAF followed by PMA/ionomycin stimulation (created with Biorender.com). (E) Quantification of NK cell markers of cytotoxicity: CD107a, granzyme B, IFN-γ, and TNF-α, as percentage of NK cells. n = 4 independent experiments; *n* = 3 for controls “NK + CAF unstimulated” and “NK + Fib unstimulated”; technical duplicates for NK + CAF or Fib stimulated; technical duplicates for NK alone, except one experiment. All data shown as mean ± SEM and statistical significance determined with one way ANOVA with Tukey’s multiple comparison test for *p* values ≤0.05. Relevant statistics shown.

To investigate if pancreatic CAFs were mediators of functional suppression of NK cells, we co-cultured the mM1 cell line with KPC-isolated CAFs or matching non-tumor-associated fibroblasts (Fibs) and pre-activated NK cells (Figure 4A). A decrease in the percentage of activating receptor DNAM-1 was noted on NK cells when cultured with CAFs (Figure 4B, Figures S4A and S4B). In contrast, activating receptor NKG2D was downmodulated on NK cells in all co-cultures, while PD-1 was upregulated, suggesting potential receptor-ligand interactions with all cell lines (Figures 4B; Figures S4A and S4B). Tumor cell death was increased when exposed to activated NK cells but impaired in the presence of either CAFs or Fibs (Figures 4C; Figure S4C). Of note, CAF and Fib cell death was minimal in the presence of NK cells in these conditions (Figure S4D).

We then addressed how CAFs or Fibs could modulate NK cell cytotoxic functions, measuring degranulation (CD107a expression), cytotoxicity (granzyme B), and cytokine release (TNF-α and IFN-γ) after phorbol-12-myristate-13-acetate (PMA)/ionomycin stimulation (Figure 4D-E; Figures S4E–S4G). Contact with CAFs decreased the frequency of NK cells expressing the degranulation marker CD107a, granzyme B and TNF-α, with no effects on IFN-γ (Figure 4E). This potent inhibitory effect was specific to pancreatic CAF co-cultures, suggesting that they have an intrinsic capacity to inhibit NK cell functions via direct contact, unlike Fibs. Nevertheless, the presence of Fibs in mM1 co-cultures led to a similar decrease in mM1 killing as CAFs, potentially a decoy-like effect via NKG2D, as Fibs also downmodulated this marker on the surface of NK cells (Figure 4B). To investigate this, we profiled mM1, CAFs, and Fibs for NK cell ligands RAE-1 (NKG2D ligand) and CD155 and Nectin-2 (DNAM-1 ligands) (Figures S5A–S5C). Fibs displayed the highest expression of RAE-1 (Figures S5A–S5C), supporting this decoy-like effect. Additionally, by treating NK cells with blocking antibodies to NKG2D or DNAM-1 in similar killing assays, we observe that mM1 killing was dependent on NK cell/tumor interactions via NKG2D and partially DNAM-1 (Figure S5D). This suggests that interference with these interactions could lead to reduced killing. It should also be considered that in tricultures, mM1 tumor cells could potentially influence the functional features of Fibs to escape NK-targeting, conferring them with CAF-like properties.

### TGF-β drives fibroblasts to suppress NK cell anti-tumor functions

**Figure 5:**
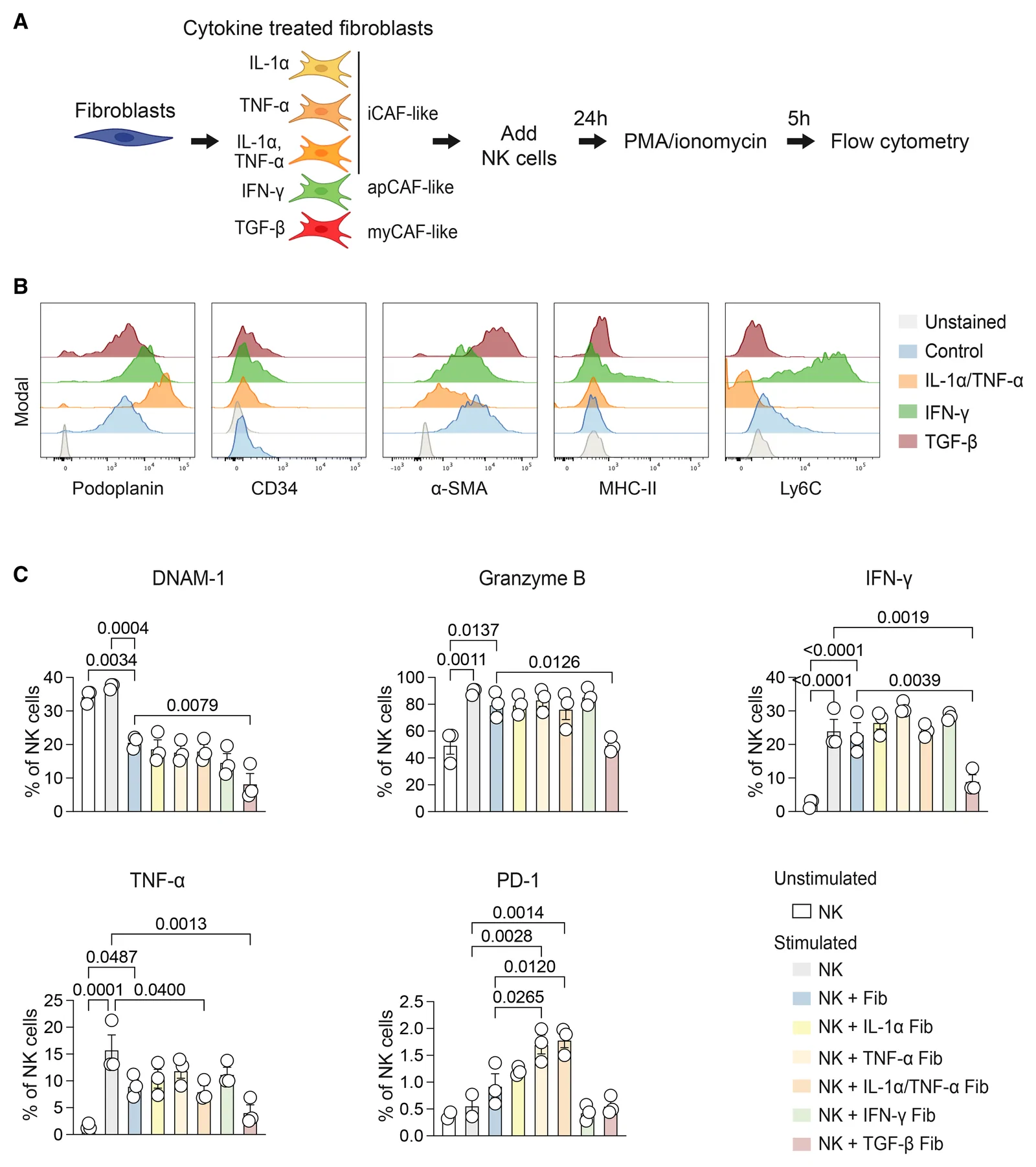
TGF-β drives fibroblasts to suppress NK cell anti-tumor functions (A)Illustration of method to generate fibroblast subtypes followed by co-culture with NK cells (created with Biorender.com). (B) Flow cytometry histograms showing the relative expression of CAF markers in fibroblasts treated with respective cytokines. (C)Flow cytometry quantification of NK cell DNAM-1, granzyme B, IFN-γ, TNF-α, and PD-1 as percentage of NK cells. n = 3 independent experiments. All data shown as mean ± SEM and statistical significance determined with one-way ANOVA with Tukey’s multiple comparison test for *p* values ≤0.05. Relevant statistics shown.

While observing an inhibitory role for pancreatic CAFs on NK cell functions, this cell line contains a mix of CAF subpopulations and myofibroblastic features typically driven by monolayer culture,^13^ thus, not discriminating between CAF subtypes. To interrogate if different CAF-like states present in the TME exert distinct NK cell modulatory capacity, we sought to investigate which signals drive fibroblasts to inhibit NK cells. We isolated fibroblasts and cultured them to a maximum of 7 days prior to cytokine treatments. IL-1α, TNF-α, or both were used to promote an iCAF-like phenotype,^13^ IFN-γ to induce apCAF-like features^11,40^ and TGF-β to induce my-CAF-like cells, as previously reported.^13,40–42^ We then tested the role of these subpopulations on mediating NK cell functions using PMA/ionomycin stimulation (Figure 5A). The respective CAF-like phenotypes after cytokine treatment were confirmed by flow cytometry and qPCR, analyzing typical markers (Figures 5B; Figures S6A–S6C). After stimulation in the presence of TGF-β-myCAF-like CAFs, fewer NK cells expressed activator receptors, cytotoxicity markers and inflammatory cytokines, including DNAM-1, granzyme B, IFN-γ, and TNF-α (Figure 5C). In contrast, control fibro-blasts, IL-1α, TNF-α-iCAF-like, and IFN-γ-apCAF-like CAFs only significantly downmodulated DNAM-1 on NK cells (Figure 5C), having no effect on granzyme B and IFN-γ expression (Figure 5C). IL-1α/TNF-α fibroblasts modestly downregulated TNF-α expression on NK cells (Figure 5C). Interestingly, TNF-α-iCAF-like were the only inducers of PD-1 expression on NK cells (Figure 5C). Overall, distinct roles for fibroblast sub-populations on NK cell functions were identified in this *in vitro* system. TGF-β primed-fibroblasts induced a strong downmodulation of NK cell cytotoxic functions, similar to the CAF cell line, pointing to a pro-tumor role of myCAF. While direct contact induced this potent inhibition, myCAF also express TGF-β in an autocrine mode, potentially synergizing in NK cell inhibition via this axis.^43^

To validate these findings, we isolated and cultured iCAF and myCAF from tumors and assessed their direct impact on NK cell functions. While tumor organoids cultured in Matrigel can drive PSCs (pancreatic stellate cells) into CAF subtypes found in tumors,^12,13^ this system would not discriminate if potential phenotypic changes in NK cells would be tumor or CAF-driven. To overcome confounding effects, we implanted mM1 into CA-G.EGFP:αSMA-RFP reporter mice, generating tumors where all CAF would be GFP^+^, and would express RFP upon αSMA upregulation. Here, PDPN/CD34/αSMA-RFP discriminates iCAF from myCAF (Figure S7A). iCAF and myCAF were cultured in a collagen/GelMa hydrogel mix^44^ to maintain the iCAF phenotype in culture: i.e., no αSMA-RFP upregulation (Figure S7B). myCAF could not be sustained under this hydrogel system (Figure S7B), perhaps not surprisingly, as they require direct contact with tumor cells^12^ and a stiff environment. Therefore, the α-SMA-expressing Pan-CAF cell line described was used as a control for NK cell functional suppression. Despite close contacts between NK cells and iCAFs observed by microscopy, and akin to the IL-1α/TNF-α-iCAF-like cells, primary iCAF did not significantly impact NK cell functions (Figures S7B and S7C). Interestingly, Pan-CAF cell lines maintained in these gels lost some of the NK cell suppressing properties demonstrated before, indicating possible mechanically mediated plasticity in immune modulatory capacity, but they still induced DNAM-1 and TNF-α downmodulation and PD-1 upregulation on NK cells (Figure S7C). These results suggest that CAFs in less mechanically stiff environments and iCAFs do not contribute to downregulation of NK cell cytotoxicity as strongly as TGF-β driven myCAFs, despite other interactions still at play, such as DNAM-1 downmodulation.

### NK cells migrate toward fibroblast-derived factors

**Figure 6:**
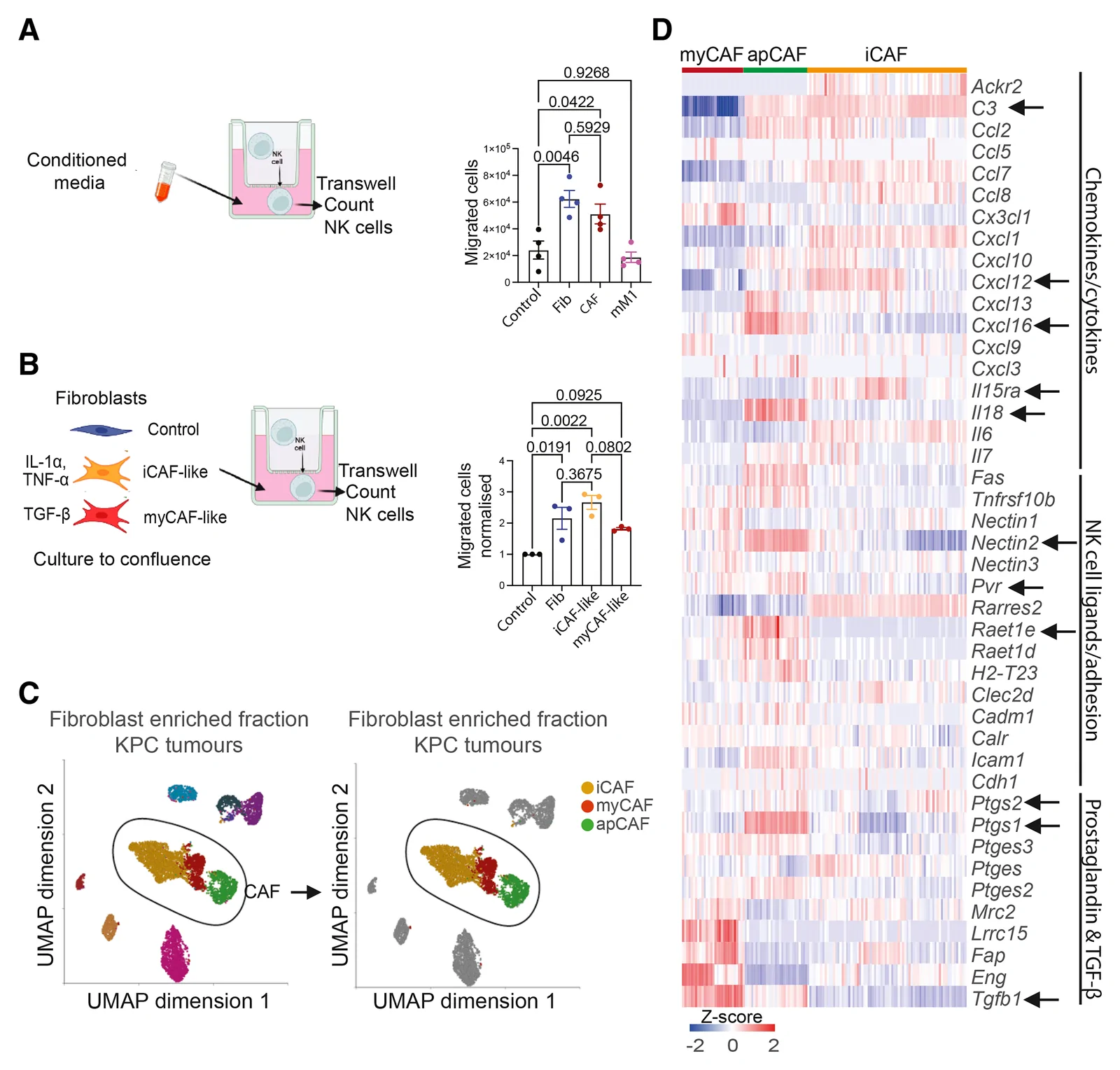
NK cells migrate toward fibroblast and CAF derived factors associating with NK-attracting chemokine signatures expressed across CAF subpopulations in pancreatic tumors (A) Transwell migration assays showing NK cell counts after migration toward respective conditioned media. n = 4 independent experiments. (B) Transwell migration assays showing NK cell counts by flow cytometry (gating on Figure S8A) after migration toward respective fibroblast subtypes and control medium, *n* = 3. NK cell counts normalized to the respective condition. All data shown as mean ± SEM and statistical significance determined with one-way ANOVA with Tukey’s multiple comparison test for *p* values ≤0.05. Relevant statistics shown. (C and D) Analysis of a publicly available single-cell RNA sequencing dataset consisting of a fibroblast-enriched fraction from KPC tumors (GEO: GSE129455).^11^ (C) UMAP plot of single-cell relative gene expression analysis (8,523 individual cells) highlighting fibroblast clusters identified.^11^ (D) Heatmap of relative gene expression from a pre-defined list of NK cell related CAF markers (Table S1), comparing its expression between CAF subtypes (each row represents a single cell). Arrows highlight markers of interest between CAF subtypes (ANOVA results on Table S1).

Despite significantly impacting NK cell functions *in vitro*, my-CAFs were not frequently identified in close interactions with NK cells in both murine tumors and human PDAC. To test if CAFs had distinct NK cell homing properties, we performed transwell migration assays with conditioned media. This confirmed that NK cells migrate toward both non-tumor pancreatic fibroblasts (Fibs) and CAF-derived factors but not toward tumor-derived factors (Figure 6A). To interrogate if this could be CAF subpopulation specific, we generated iCAF and myCAF-like fibroblasts as before and cultured them to confluence in a transwell system (Figure 6B). When measuring NK cell migration toward the distinct CAF-like populations by flow cytometry, we confirmed that NK cells are capable of migration toward fibroblasts and iCAF-like fibroblasts but migrated significantly less toward TGF-β-like fibroblasts (Figures 6B; Figure S8A).

To investigate which mechanisms could underlie the different patterns of NK cell localization in tumors, we analyzed publicly available single-cell gene expression data from the fibroblast-enriched fraction from autochthonous murine KPC tumors (GEO: GSE129455).^11^ Uniform manifold approximation and projection (UMAP) analysis of the fibroblast-enriched fraction from KPC tumors identified 3 major fibroblast clusters (Figure 6C). To discriminate between iCAF, myCAF, and apCAF, we used typical fibroblast markers described previously^11^ (Figure S8B). Next, we defined a list of CAF markers with potential to influence NK cell behavior (Table S1). iCAFs and apCAFs expressed higher levels of several chemokines including *Cxcl12*, a potent chemo-attractant and suppressor of NK cells^45^ (Figure 6D), whereas my-CAF displayed a more restricted chemokine expression pattern, with decreased *Cxcl12* expression, only expressing higher levels of *Cx3cl1* (Figure 6D). In contrast to myCAF, iCAF and apCAF expressed *Il15ra* and *Il18*, which can sustain and activate NK cells intratumorally^46^ (Figure 6D). Strikingly, iCAFs and apCAFs expressed more *C3* than myCAFs, and it is known that C3b can inhibit NK cells when binding to NK receptor CD11b.^47^ DNAM-1 and NKG2D ligands were also expressed across CAF subtypes, with a higher expression of *Pvr* and *Nectin2* by apCAF (Figure 6D, Table S1). As anticipated, myCAF expressed diverse collagen and ECM genes and the highest levels of TGF-β pathway-associated genes, thus harboring the potential to sup-press NK cells via these axes (Figure 6D, Table S1). Lastly, genes encoding PG synthesis enzymes, such as *Ptgs2*, were expressed across all CAF subtypes, suggesting an additional layer of potential NK cell suppression.^31^ Altogether, these results reveal that in addition to their high capacity for suppressing NK cell cytotoxicity, myCAFs are less able to attract and sustain NK cells intratumorally, correlating with NK cell exclusion from these stromal areas.

### Targeting PGE2 partially rescues NK cell cytotoxicity

**Figure 7:**
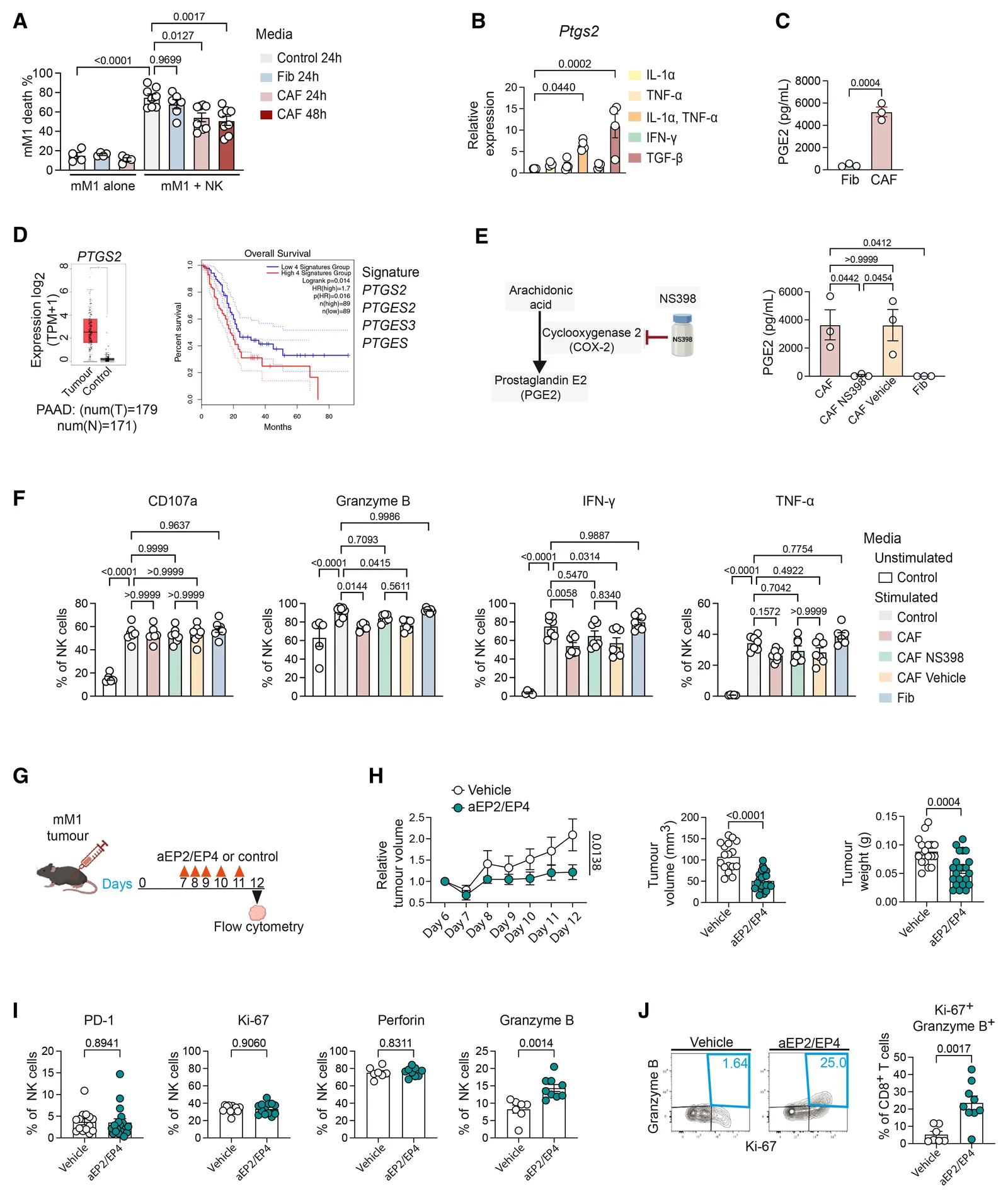
Pancreatic CAF-derived soluble factors including PGE2 inhibit NK cell functions (A)Flow cytometry quantification of NK-mediated mM1 tumor death under conditioned media treatment (gating on Figure S9A). n = 4 independent experiments (duplicates for conditions with NK cells). (B) Relative expression of *Ptgs2* in fibroblasts primed with different cytokines by RT-qPCR as 2 ^-ΔΔCT using *Gapdh* as reference gene and fibroblasts as reference sample. n = 4 independent cultures. (C)PGE2 ELISA comparing pancreatic Fib versus CAF-conditioned media; *n* = 3 media batches. (D) Analysis of publicly available datasets from TCGA and GTEx databases using Gepia2.^51^ *PTGS2* expression between healthy and patients with pancreatic adenocarcinoma. Statistical differences determined with *t* test for *p* value ≤0.01. Kaplan–Meier curves showing overall survival of patients with pancreatic adenocarcinoma with high vs. low expression of *PTGS2, PTGES3, PTGES*, and *PTGES2* combined signature.^51^ Statistical significance determined with log -rank test for *p* ≤ 0.05. (E)Illustration of NS398 mode of action (created with Biorender.com) and ELISA of PGE2 of CAF, NS398-treated, and vehicle control CAF-derived and Fib conditioned media. n = 3 batches. (F) Flow cytometry quantification of NK cell cytotoxicity in the presence of conditioned media blocking the PGE2 pathway (CD107a, granzyme B, IFN-γ, and TNF-α). n = 4 independent experiments; technical duplicates when possible. (G) Illustration of mM1 tumor model under EP2/EP4 antagonism (created with Biorender.com). (H) Relative tumor volumes (relative to first measurement), endpoint tumor volumes, and weights. 4 independent experiments, *n* = 17 (vehicle control), *n* = 19 (antagonists of EP2/EP4 or aEP2/EP4). 2 mice excluded as tumors did not develop. Samples were excluded for flow cytometry analysis applying similar criteria (if insufficient material for analysis). (I) Flow cytometry NK cell functional profile, comparing vehicle with aEP2/EP4. As percentage of NK cells: PD-1, Ki-67, perforin, and granzyme B. (J) Flow cytometry gating and quantification of Ki-67^+^ granzyme B^+^ CD8^+^ T cells comparing control with aEP2/EP4. 3 experiments pooled for PD-1 and Ki-67 analysis, *n* = 10 and *n* = 13 for vehicle and aEP2/EP4, respectively. 2 experiments pooled for granzyme B and perforin panel: *n* = 7 (vehicle), *n* = 9 (aEP2/EP4). All data shown as mean ± SEM and statistical significance determined with one-way ANOVA with Tukey’s multiple comparison, two-way ANOVA with Šídák’s multiple comparisons (for tumor volumes during tumor progression) or two-tailed unpaired *t* test for *p* values ≤0.05.

Next, we investigated if CAF-derived soluble factors could mediate the direct suppression of NK cells. We used the KPC-matched cell lines described to generate conditioned media. CAF-derived soluble factors reduced the ability of NK cells to kill mM1 tumor cells in contrast to Fib-conditioned medium (Figure 7A; Figure S9A). From gene expression data (Figure 6D) and mass spectrometry data from pancreatic CAF vs. Fib secretome^48^ (Figure S9B), we selected potential targets including ECM proteins^49^ and regulators of PGE2.^31,50^ Treatment with recombinant laminin, perlecan, and osteopontin ECM proteins did not impact NK cell killing of tumor cells (Figure S9C). However, genes associated with the PGE2 synthesis pathway, such as *Ptgs2, Ptges2*, and *Ptges* were expressed across distinct CAF subtypes (Figures 6D; Table S1). Furthermore, *Ptgs2*, encoding cyclooxygenase-2 (COX-2), was upregulated in TGF-β-myCAF-like fibroblasts and IL-1α/TNF-α-like iCAFs (Figure 7B). Importantly, ELISA revealed significantly higher levels of PGE2 in CAF-conditioned medium compared to Fib-derived medium (Figure 7C). To address if the PGE2 pathway could be relevant in human PDAC, we assessed TCGA and GTEx databases using Gepia2.^51^ *PTGS2*, encoding COX-2, was significantly upregulated in patient-derived samples compared to control, similar to *PTGES2, PTGES3*, and *PTGES*, encoding for PGE2 synthases (Figure 7D; Figure S9D). The combined signatures of PGE2 pathway enzymes associated with worse overall survival (Figure 7D; Figure S9D).

COX-2 is required for the enzymatic conversion of arachidonic acid to PGs, and it can be blocked with the COX-2 inhibitor NS398 thereby preventing PG production^52^ (Figure 7E). NS398 was effective at decreasing PGE2 levels in CAF-conditioned medium (Figure 7E). To determine if specific NK cell cytotoxic functions were restored by NS398 treatment of CAFs, we analyzed NK cell cytotoxic profile under conditioned media treatment followed by stimulation with PMA/ionomycin. Although there were no significant changes in CD107a and TNF-α expression (Figure 7F), both granzyme B and IFN-γ were partially restored with NS398-CAF medium, as their levels of expression were downmodulated by CAF and vehicle control media but not by NS38-CAF medium (Figures 7F; Figures S9E and S9F).

To first determine if tumor-associated NK cells can control tumor growth, we performed NK cell depletion experiments using an anti-NK1.1 antibody in the mM1 model (Figure S10A). Tumor growth was similar between control and NK cell depleted tumors, but a coincident increase in the frequency of granzyme B^+^ CD8^+^ T cells was observed when NK cells were depleted (Figures S10B–S10D). This suggests that dysfunctional tumor-associated NK cells drive CD8^+^ T cell inhibition, and their removal allows for T cell cytotoxic function. To determine if targeting PGE2 activity *in vivo* could improve intratumoral NK cell cytotoxic profile, rather than disrupting PGE2, we inhibited the PGE2 receptors EP2 and EP4 with selective antagonists that have been shown to more specifically regulate NK cell functions.^53^ EP2/EP4 antagonism (aEP2/EP4) slowed tumor growth in the mM1 model (Figures 7G and 7H). Immune phenotyping of control and aEP2/EP4 tumors showed no differences in terms of myeloid, T, NK, and NKT cell frequency (Figure S11A and S11B). However, in treated tumors, the frequency of granzyme B-expressing NK cells and CD8^+^ T cells was significantly increased, as was CD8^+^ T cell proliferation (Figure 6I-J; Figures S11C and S11D). By contrast, PD-1 and perforin expression remained unchanged in NK and CD8^+^T cells (Figures 7I; Figure S11D) as was the proportion of immature non-cytotoxic, NKG2A^+^, Ly49H^−^ NK cells (Figures S12A and S12B). Overall, and despite the potential of PGE2 to be used as a general CAF target in PDAC, EP2/EP4 antagonism did not reverse the non-cytotoxic CD11b^−^ NKG2A^+^ Ly49H^−^ tumor-associated NK cell profile, pointing to the importance of targeting multiple suppressive axes in combinational therapy to unleash both NK and T cell functions intratumorally.

## Discussion

Owing to their cytotoxic properties and “off-the-shelf” delivery potential, NK cells are increasingly exploited for cancer therapy.^21^ As previously reported, we observed that NK cells were rare in human PDAC.^28^ They predominantly located in the stroma at invasive tumor margins, where less desmoplasia is detected^12,54^ and tumor budding can occur,^55^ similar to breast cancer studies.^56^ NK cells closely associated with PDGFR-α^+^/PDPN^+^ CAFs in murine PDAC samples and less frequently near α-SMA^+^ CAFs, corroborating NK cell exclusion from these areas in human PDAC. These observations mirror findings where CD8^+^ T cells were excluded from central desmoplastic areas in PDAC.^57^ In line with this, transwell assays indicated that pancreatic fibroblasts and mixed CAF populations attract NK cells, but TGF-β-myCAF-like fibroblasts promoted NK cell migration less efficiently. In contrast, iCAF-like fibroblasts strongly induced NK migration while both iCAF and apCAF expressed a range of chemokines linked to NK homing. Nonetheless, NK cells did not closely associate with Ly6C^+^ PDPN^+^ iCAFs in autochthonous KPC tumors, suggesting that other PDPN^+^/PDGFR-α^+^ CAF sub-populations contribute to NK cell recruitment, or that *in vitro* generated iCAFs possess a distinct secretome from *in situ* iCAFs. It is also possible that NK cells face other barriers such as interaction with myeloid cells, restricting localization to iCAF-rich areas. iCAF from autochthonous KPC tumors also differentially overexpress *Il15ra*, which NK cells are dependent upon^46^ and apCAF express NK cell-directed chemokines and *Il18*,^46^ suggesting that both could provide intratumoral NK cell support. While myCAF overexpressed *Cx3cl1* versus iCAF and apCAF, this was the only NK-chemokine they upregulated. Additionally, both pancreatic fibroblasts and CAFs expressed ligands for NKG2D and DNAM-1 on NK cells, similar to reports in breast cancer.^56^ This expression and the decoy-like effects observed in killing assays point to additional axes of communication between CAFs and NK cells, likely influencing their intratumoral position and function.

Although we did not map NK cell cytotoxicity/maturation to intratumoral localization, most NK cells displayed an immature/ non-cytotoxic phenotype that became dominant as tumors progressed. A similar pattern was observed in human PDAC, where NK cells were predominantly CD56^bright^, a subset associated with poor cytotoxicity.^35^ *In vivo*, intratumoral NK cells differed markedly from circulating counterparts, exhibiting downregulation of CD11b, suggesting a tumor-induced shift toward immature, non-cytotoxic states. Indeed, conversion to a dysfunctional phenotype has been linked to rapid CD11b downmodulation.^58^ Also consistent with our data, poorly cytotoxic CD11b^–^ CD27^–^ NK cells were identified in liver cancer prevalent in late-stage tumors.^59^ Interestingly, iCAFs and apCAFs expressed markedly higher levels of C3 than myCAFs. C3 encodes C3b, a potent suppressor of NK cell cytotoxicity upon binding CD11b.^47^ While CD11b downmodulation could point to this interaction, further investigation is needed. We also noted a lack of activation receptor Ly49H on intratumoral NK cells. Ly49H marks long-lived memory NK cells which recognize and quickly respond to mouse cytomegalovirus (MCMV) re-activation.^60^ While the role of Ly49H in solid tumors is unclear, in patients with leukemia undergoing hematopoietic stem cell transplant, expansion of NK cells expressing human homolog NKG2C associates with reduced relapse.^61^ Whether Ly49H^−^ NK cells express receptors which would influence their extravasation into tumors remains to be determined. We further observed higher NKG2A expression compared with circulating NK cells, previously reported as an immune evasion mechanism in metastatic PDAC.^62^ PD-1 was also upregulated *in vivo*, albeit in a minority of NK cells. *In vitro*, PD-1 upregulation was detected in the presence of iCAF-like cells, pointing to the possibility of CAF modulation via this axis. In functional assays, we found that pancreatic CAFs display an intrinsic capacity to inhibit NK cells, with TGF-β-driven myCAF-like being strong suppressors of NK cytotoxicity. In contrast, non-tumor fibroblasts, iCAF-like and tumor-derived iCAF lacked this intrinsic suppressive behavior. Moreover, and illustrating CAF plasticity, softer environments like hydrogels decreased CAF suppressive capacity, corroborating findings where senescent myCAF which secrete stiff ECM induced higher inhibition of NK cells in breast cancer.^63^ Together, our results point to myCAF as a dominant suppressor of NK cells, contrasting with reported tumor-restraining roles of myCAF.^64,65^ Nevertheless, heterogeneity within the larger myCAF umbrella does exist. For example, CD105,^41^ Endo180,^66^ LRRC15,^67^ and senescent p16^63,68^ mark tumor-promoting α-SMA^+^ myCAF. While other CAF dichotomies described in PDAC such as FAP^+^ vs. α-SMA^−^CAF were not explored here,^69–71^ targeting α-SMA^+^ CAF in general should be avoided, due to tumor-restraining roles being lost.^64,65^ Instead, approaches to restrict tumor-promoting myCAF could be considered; for instance, TGF-β signaling was identified as the main tumor-promoting setpoint in these myCAF,^41,66,67^ potentially being a target of interest with implications on NK cells. Interestingly, a PDAC study identified netrin G1 as a marker expressed across CAFs which enhances their cytokine release compared to cancer-adjacent fibroblasts; netrin G1^+^ CAFs were shown to secrete high levels of TGF-β, while its abrogation led to decreased TGF-β and restoration of IL-15, thus rescuing NK killing capacity.^34^ While the authors did not discriminate CAF sub-populations, it would be interesting to integrate netrin G1 expression within the myCAF landscape and NK cell niches.

Non-tumor pancreatic fibroblasts had no detectable impact on NK cell cytotoxicity per se, but they significantly decreased NK cell-mediated killing of tumor cells, highlighting additional axes of tumor evasion, potentially via NKG2D or DNAM-1. While this could draw a parallel of suppression by iCAF/apCAF, it also needs to be considered that iCAF might recruit myeloid cells and thus promote indirect NK cell suppression and exclusion.^33^

Having determined distinct roles for CAF states in modulating NK cell recruitment and functions, we also detected expression of NK suppressive genes across CAF subpopulations, and set to investigate if CAF-derived soluble factors could be therapeutically targeted. Despite enhanced myCAF deposition of ECM, laminin, perlecan and osteopontin did not module NK behavior in our hands. While we did not investigate how senescent myCAFs impact NK cell functions, others report a significant role for ECM derived from senescent myCAF in suppressing NK cells.^63^ We identified the PGE2 pathway as a relevant NK cell target, influencing granzyme B expression. However, EP2/EP4 antagonism did not alter frequencies of immature tumor-infiltrating CD11b^−^ NKG2A^+^ Ly49H^−^ NK cells, suggesting that additional CAF-NK axes may need to be targeted to fully restore NK cell cytotoxicity. This might be of consideration in the context of restoring CD8^+^ T cell cytotoxicity, as NK cell depletion experiments point to a role for dysfunctional intratumoral NK cells in suppressing CD8^+^ T cells. Importantly, PGE2 is also expressed by tumor cells in PDAC,^72^ with potential to fuel suppressive macrophages,^73^ a mechanism that could also be at play, despite not on the scope of this investigation.

Overall, our study revealed previously unappreciated, distinct roles of CAF subtypes in modulation of NK anti-tumor functions which could have implications for NK cell-based therapy for solid tumors.

### Limitations of the study

We did not investigate the role of myCAF subsets such as senescent myCAF in regulating NK cell function. Although CAF heterogeneity was retained in the syngeneic mM1 model, we are aware of its limitations in representing pancreas-specific features. To address this, we imaged autochthonous KPC tumors and analyzed a public gene expression dataset to further characterize NK cell infiltration. While few human PDAC samples were available for flow cytometric profiling, murine models mirrored the accumulation of poorly cytotoxic/immature NK cells observed in human PDAC. Another limitation is that we could not selectively perturb CAF subsets *in vivo* because of CAF plasticity and lack of subset-specific Cre lines. We also did not assess EP2/EP4 antagonism in the autochthonous model, which would better reflect PDAC biology. Lastly, although EP2/EP4 targeting is more specific than global PGE2 blockade, the pathway was not selectively inhibited in NK cells, preventing us from distinguishing direct effects on T cells from those mediated via NK cell modulation.

## Star★Methods

Detailed methods are provided in the online version of this paper and include the following:

- KEY RESOURCES TABLE
- EXPERIMENTAL MODEL AND STUDY PARTICIPANT DETAILS
  ○ Human PDAC samples
  ○ Mice
  ○ Cell lines and pancreatic CAFs and Fibs
- METHOD DETAILS
  ○ Ethics statement for human PDAC samples
  ○ Processing of human PDAC tumors
  ○ Immunohistochemistry and H&E of human PDAC samples
  ○ Immunofluorescence (IF) of human PDAC tumor microarray (TMA)
  ○ Cell lines and pancreatic CAFs and Fibs culture
  ○ Murine tumors and tissue processing
  ○ *Ex vivo* culture of iCAF in collagen/GelMa hydrogels
  ○ Primary NK cells isolation and culture
  ○ Primary fibroblasts isolation and culture
  ○ Generation of conditioned media
  ○ Killing assays
  ○ NK cell degranulation and cytokine release assays
  ○ Transwell migration assays
  ○ Flow cytometry
  ○ Immunofluorescence imaging of murine tumors
  ○ Enzyme-linked immunosorbent assays (ELISA)
  ○ RNA extraction and one step RT-qPCR
  ○ Analysis of cell-to-cell distances in murine mM1 tumors
  ○ Analysis of publicly available datasets
- QUANTIFICATION AND STATISTICAL ANALYSIS

## Star★Methods

### Key Resources Table

**Table:** 

REAGENT or RESOURCE	SOURCE	IDENTIFIER
Antibodies		
Anti human CD56	Dako Agilent	Autostainer Link 48, Clone 123C3; RRID:AB_2750583
Anti human α-SMA	Abcam	Cat# ab150301; RRID:AB_3675461
Anti human NCAM-1/CD56	Novus Biologics	Cat# AF2408; RRID:AB_442152
Anti human PDGFR-α	R&D Systems	Cat# AF-307-NA; RRID:AB_354459
Anti-mouse PDGFR-α	R&D Systems	Cat# AF1062; RRID:AB_2236897
Anti mouse perlecan,	Invitrogen	Cat# MA1-06821; RRID:AB_559394
Anti mouse α-SMA	Invitrogen	Cat# 41-9760-82; RRID:AB_2573631
Anti mouse Klrb1c/CD161c (NK1.1)	Cell Signaling	Cat# 39197; RRID:AB_2892989
Anti mouse podoplanin	Biolegend	Cat# 127404; RRID:AB_1134222
Anti mouse CK19	Merck	Cat# MABT913; RRID:AB_2892523
Anti mouse Ly6C	Abcam	Cat# AB54223; RRID:AB_881384
Fc block 1:300	BioXCell	Cat# CUS-HB-197, clone 2.4G2, RRID:AB_2687830
Fixable viability dye	Invitrogen	65-0865-18
Fixable viability dye	Invitrogen	L34955
LIVE/DEAD^™^ Fixable Blue dye	Invitrogen	L23105A
Anti mouse CD107a	Biolegend	PE-Cy7, clone 1D4B; RRID: AB_2562146
Anti mouse CD11b	Biolegend	APC-Cy7 or FITC, clone M1/70; RRID: AB_830641 and AB_312788
Anti mouse CD11c	Biolegend	BV605, clone N418; RRID:AB_11204403
Anti mouse NKG2A	Biolegend	APC or PE-Cy7, clone 16A11; RRID: AB_11125166 and AB_2728160
Anti mouse CD27	Biolegend	PercPCy5.5, BV785 or BV421, clone LG.3A10; RRID: AB_2073424, AB_2800595 and AB_2565547
Anti mouse CD3	Biolegend	AF488 or BV785, clone 17A2; RRID: AB_493530 and AB_11218805
Anti mouse CD31	Biolegend	BV785 (clone 390), FITC and PercPCy5.5 (clone MEC13.3); RRID: AB_2810334, AB_312913 and AB_2566761
Anti mouse CD34	Biolegend	BV421, clone MEC14.7; RRID: AB_312922
Anti mouse CD4	Biolegend	PercPCy5.5, clone GM1.5; RRID: AB_312690
Anti mouse CD45	Biolegend	BV785, APC or BV510, clone 30-F11; RRID: AB_2564590, AB_312976 and AB_2561392
Anti mouse CD8a	Biolegend	BV785, clone 53-6.7; RRID: AB_11218801
Anti mouse DNAM-1	Biolegend	PE-Cy7 or PercPCy5.5, clone TX42.1; RRID: AB_2716223 and AB_2716085
Anti mouse Granzyme B	Biolegend	PercPCy5.5, clone QA16A02; RRID: AB_2728378
Anti mouse IFN-γ	Biolegend	BV421 or BV785, clone XMG1.2; RRID: AB_10897937 and AB_11219004
Anti mouse Ly49H	Biolegend	APC, clone 3D10; RRID: AB_2783110
Anti mouse Ly6C	Biolegend	APC, clone 3D10; RRID: AB_2783110
Anti mouse NK1.1	Biolegend	PE or BV421, clone PK136; RRID: AB_313394 and AB_10895916
Anti mouse NKp46	Biolegend	PE, clone 29A1.4; RRID: AB_10552741
Anti mouse PD-1	Biolegend	APC-Cy7 or BV605, clone 29F.1A12; RRID: AB_2563522 and AB_11125371
Anti mouse PDGFR-α	Biolegend	PE-Cy7, clone APA5; RRID: AB_2715973
Anti mouse Perforin	Biolegend	APC, clone S16009B; RRID: AB_2721464
Anti mouse Podoplanin	Biolegend	APC-Cy7 or PE-Cy7, clone 8.1.1; RRID: AB_2629803 and AB_10613294
Anti mouse TNF-α	Biolegend	FITC, clone MP6-XT22; RRID: AB_315424
Anti mouse CD155	Biolegend	PE-Cy7, clone TX56; RRID: AB_2564298
Anti mouse CD49b	BD Biosciences	BV510, clone HMα2; RRID: AB_2739890
Anti mouse NK1.1	BD Biosciences	BUV395, clone PK136; RRID: AB_2738618
Anti mouse TIGIT	BD Biosciences	BV786, clone 1G9; RRID: AB_2742064
Anti mouse CD3	BD Biosciences	FITC, clone 17A2; RRID: AB_395698
Anti mouse CD34	eBioscience™	eFluor660, clone RAM34; RRID: AB_10596826
Anti mouse FoxP3	eBioscience™	eFluor450, clone FJK-16s; RRID: AB_1518812
Anti mouse Granzyme B	eBioscience™	PE, clone NGZB; RRID: AB_10870787
Anti mouse KI-67	eBioscience™	PE-Cy7, clone SolA15; RRID: AB_11220070
Anti mouse MHC-II-I-A/I-E	eBioscience™	eFluor450, clone M5/114.15.2; RRID: AB_467561
Anti mouse NKG2D	eBioscience™	PE-Cy7 or PE, clone CX5; RRID: AB_469657 and AB_465996
Anti mouse Perforin	eBioscience™	APC, clone eBioOMAK-D; RRID: AB_469514
Anti mouse α-SMA	eBioscience™	eFluor570, clone 1A4; RRID: AB_2573631
Anti mouse RAE-1	R&D Systems	AF647, clone 199205; RRID: AB_3647105
And mouseCD155	Biolegend	PE-Cy7, clone TX56; RRID: AB_2564298
Anti human CD45	Biolegend	FITC, clone HI30; RRID: AB_314393
Anti human CD3	Biolegend	BV421, clone OKT3; RRID: AB_2565848
Anti human NKp46	Biolegend	BV785, clone 9E2; RRID: AB_2810508
Anti human CD56	Biolegend	APC/Fire^™^ 750, clone 5.1H11; RRID: AB_257210
Anti human CD16	Biolegend	PercP, clone 3G8; RRID: AB_940378
Anti human CD11b	BD Biosciences	BUV496, clone D12; RRID: AB_2874280
Anti mouse DNAM-1	eBioscience^™^	Cat# 16-2261-81, clone 10E5; RRID: AB_1724031
Rat IgG2b kappa isotype control	eBioscience^™^	Cat# 16-4031-81, clone eB149/10H5; RRID: AB_470151
Anti mouse NKG2D	R&D Systems	Cat# MAB1547, clone 191004; RRID: AB_2133391
Rat IgG2b isotype control	R&D Systems	Cat# MAB0061, clone 141945; RRID: AB_357350
NK1.1 mAb clone	BioXCell	Cat# BE0036, clone PK136; RRID: AB_1107737
Mouse IgG2a, k	BioXCell	Cat# BE0085, clone C1.18.4; RRID: AB_1107771
Biological samples		
Human PDAC TMA	Biomax	PA807a
Human PDAC samples (see ethics statement)	NA	Tissue bank of the University Medical Center Mainz
Chemicals, peptides, and recombinant proteins		
Collagenase A	Roche	10103578001
Collagenase D	Roche	11088858001
DNAse	Roche	03724778103
Phorbol-12-myristate-13-acetate (PMA)	Sigma	P8139
Ionomycin	Abcam	ab120370
1 × eBioscience^™^ Cell Stimulation Cocktail	Invitrogen	00-4970-93
Mouse perlecan	Sigma	H4777
Mouse osteopontin	R&D Systems	441-OP-050
Mouse laminin	Invitrogen	23017-015
Murine IL-2	Peprotech	212-012
CellTrace™ CFSE	Invitrogen	C34554
CellTrace^™^ Far Red	Invitrogen	C34564
Murine IL-1α	Peprotech	211-11A
TNF-α	Peprotech	315-01A
IFN-γ	Peprotech	315-05
TGF-β	Peprotech	34-8342-85
EP2 antagonist	Cayman Chemical	15016
EP4 antagonist	Cayman Chemical	15973
Critical commercial assays		
PGE2 ELISA kit	R&D Systems	KGE004B
Qiagen RNeasy Mini kit	Qiagen	74106
TaqMan^™^ RNA-to-CT^™^ 1-Step Kit	Applied Biosystems	4392938
Foxp3/Transcription Factor Staining Buffer Set	eBioscience™	00-5523-00
NK cell isolation kit	Miltenyi	130-115-818
Rat tail type I Collagen	Corning	354236
GelMa	Sigma	900622-1G
Photoinitiator LAP	Sigma	900889-5G
RPMI 1640 medium with L-glutamine and sodium bicarbonate	Sigma	R8758-500 mL
DMEM medium with D-glucose, L-glutamine and pyruvate	Gibco	41966-029
Experimental models: Cell lines		
B16-F10 melanoma cell line	ATCC	CRL-6475 ^™^
mM1 pancreatic tumor cell line	Professor David Tuveson, CSHL	Derived from KPC (LSL-KrasG12D/+; LSLTp53R172H/+;Pdx-1-Cre) model^36^,^74^
Murine pancreatic cancer associated fibroblasts (Pan CAF)	Tobias Janowitz	Derived from autochthonous KPC (LSL-KrasG12D/+;LSLTp53R172H/+;Pdx-1-Cre) tumors^74^
Murine non-tumor pancreatic fibroblasts (Pan Fib)	Tobias Janowitz	Derived from autochthonous KPC (LSL-KrasG12D/+;LSLTp53R172H/+;Pdx-1-Cre) tumors^74^
Experimental models: Organisms/strains		
CAG.EGFP:αSMA-RFP	Jackson laboratory strains crossed	Crossing C57BL/6-Tg(CAG-EGFP)131Osb/LeySopJ mice (strain 006567) with B6.FVB-Tg(Acta2- DsRed)1Rkl/J (strain 031159)
Oligonucleotides		
Taqman gene expression assay Ptgs2	TermoFisher Scientific	Mm00478374_m1
Taqman gene expression assay Cxcl12	TermoFisher Scientific	Mm00445553_m1
Taqman gene expression assay Cxcl10	TermoFisher Scientific	Mm00445235_m1
Taqman gene expression assay Lif	TermoFisher Scientific	Mm00434761_m1
Taqman gene expression assay Il6	TermoFisher Scientific	Mm00446190_m1
Taqman gene expression assay Acta2	TermoFisher Scientific	Mm00725412_s1
Taqman gene expression assay Gapdh	TermoFisher Scientific	Mm99999915_g1
Software and algorithms		
Fiji macro to analyze NK cell distances	NA	https://github.com/jboulanger/MultiplexTissueColocalization

### Experimental Model and Study Participant Details

#### Human PDAC samples

Human tissue samples were provided by the tissue bank of the University Medical Center Mainz in accordance with the regulations of the tissue biobank and the approval of the ethics committee of the University Medical Center Mainz. The study was approved by the ethics committee of the University of Mainz (Statement: 2019–14390; Landesärztekammer Rhineland-Palatinate), and a written informed consent was obtained from all patients. Human PDAC TMA for IF analysis is from Biomax PA807a, containing samples from 34 PDAC cases, 27 cancer adjacent pancreatic tissue and 13 normal pancreatic tissue.

#### Mice

All animal housing and experimental work was performed in Medical Research Council ARES animal facility under specific pathogen-free conditions (SPFs), at room temperature, with a 12h light-dark cycle, and mice remained socially housed. All experiments were done with the approval of the LMB Animal Welfare and Ethical Review Body (AWERB) and the UK Home Office, under the project license (PPL) P88378375 or PP3605301. Animal facility staff and members of the group performed or assisted with animal work. Female wild type C57BL/6JOla mice aged 6–12 weeks were used in this work for subcutaneous tumor implantations. For fibroblast and NK cell primary cell isolations, female and male mice aged 8–14 weeks were used. CAG.EGFP:αSMA-RFP were generated before by crossing C57BL/6-Tg(CAG-EGFP)131Osb/LeySopJ mice (strain 006567, purchased from Jackson laboratory) with B6.FVB-Tg(Acta2-DsRed)1Rkl/J (strain 031159, purchased from Jackson laboratory), generating reporter mice to be used for mM1 subcutaneous tumor implantation. Male and female mice aged 6–12 weeks were used.

#### Cell lines and pancreatic CAFs and Fibs

B16-F10 melanoma cell line (ATCC) and mM1 pancreatic tumor cell line derived from KPC (LSL-Kras^G12D/+^;LSLTp53^R172H/+^;Pdx-1-Cre) model^36,74^ (kindly provided by Professor David Tuveson, CSHL) were cultured in DMEM medium with D-glucose, L-glutamine and pyruvate (Gibco, 41966-029), supplemented with 10% FBS (Sigma) and 1% Penicillin-streptomycin (Life Technologies). Murine non-tumour pancreatic fibroblasts (Pan Fib) and pancreatic cancer associated fibroblasts (Pan CAF) were derived from autochthonous KPC (LSL-Kras^G12D/+;^LSLTp53^R172H/+;^Pdx-1-Cre) tumors and kindly provided by Tobias Janowitz.^48,74^

### Method Details

#### Ethics statement for human PDAC samples

Human tissue samples were provided by the tissue bank of the University Medical Center Mainz in accordance with the regulations of the tissue biobank and the approval of the ethics committee of the University Medical Center Mainz. The study was approved by the ethics committee of the University of Mainz (Statement: 2019–14390; Landesärztekammer Rhineland-Palatinate), and a written informed consent was obtained from all patients.

#### Processing of human PDAC tumors

Human PDAC tumors were processed by mincing the tissue into 1mm^3^ pieces in basal medium using a scalpel. The tissue fragments were then placed in a mixture of 1 mg/mL of Collagenase A, 1 mg/mL Collagenase D and 0.4 mg/mL DNase I for 45minutes-1 h. The reaction was inactivated using 10mM EDTA and the solution as well as any remaining tissue fragments were passed through a 70 μm filter. The fragments were then massaged using the soft side of the syringe plunger to release any loosely adherent cells. The cells were centrifuged at 350g for 5min and resuspended in buffer containing PBS and 0.1% BSA and 2mM EDTA to proceed to flow cytometry.

#### Immunohistochemistry and H&E of human PDAC samples

Paraffin embedded tissue of either whole PDAC tissue samples (*n* = 3) or a tissue microarray (TMA; *n* = 10 human tumor cases, each case with 4 tumor cores with a diameter of 1 mm with tissue from the center and the periphery of PDAC) cut into 3 μm thick sections, followed by deparaffinization and rehydration with xylene and different concentrations of ethanol using the Agilent automated staining system (Dako; Agilent Technologies, Inc.). Endogenous peroxidase was blocked using Dako EnVision™ Flex Peroxidase-Blocking Reagent for 5 min (Dako; Agilent Technologies, Inc.). As primary antibody, a monoclonal mouse anti human CD56 (Autostainer Link 48, Clone 123C3; Dako Agilent) was ready to use and incubated for 20 min. Antigen retrieval was performed using Dako EnVision™ Flex Target Retrieval Solution pH: 9.0 (Dako; Agilent Technologies, Inc.) and visualized by Dako EnVision™ Flex DAB+ Chromogen (Agilent Technologies, Inc.). Then a counterstaining with hematoxylin (Dako; Agilent Technologies, Inc.) was performed. Consecutive slides were stained with routine automated hematoxylin and eosin (H&E; Leica HistoCore SPECTRA ST). All slides were digitized with a NanoZoomer 2.0-HT scanner (Hamamatsu Photonics, Herrsching, Germany).

#### Immunofluorescence (IF) of human PDAC tumor microarray (TMA)

Human PDAC TMA (PA807a, Biomax) containing samples from 34 PDAC cases, 27 cancer adjacent pancreatic tissue and 13 normal pancreatic tissue were dewaxed and rehydrated. Samples underwent antigen retrieval in TRIS buffer PH9 for 15 min in the microwave. Samples were then washed 3 times in PBS, incubating 5 min per wash, and then blocked for 1h at room temperature in a humid chamber with block buffer: 3% (m/v) BSA, 2% (v/v) chicken serum, 0.1% (v/v) Tween 20, 0.3 M glycine in PBS. Primary antibodies were prepared in block buffer and incubated overnight at 4°C: rabbit anti-α-SMA (ab150301, Abcam) mouse anti NCAM-1/CD56 (Novus Biologics) both 1:200 and goat anti PDGFR-α (R&D, AF-307-NA), 1:100. The following day, sections were washed 3 times with PBS and incubated for 1h in the humid chamber at room temperature with appropriate conjugated secondary antibodies (Invitrogen, 1:300). Samples were washed 3 times in PBS and stained with DAPI solution, washed again, then mounted with ProLong Gold Antifade medium (Invitrogen, P36930). Slides were sealed with nail polish. Stained tumor sections were imaged at 20× magnification on an EVOS M7000 Imaging System. Images were processed in QuPath.

#### Cell lines and pancreatic CAFs and Fibs culture

All cells were cultured in cell incubators at 37°C, 5% CO_2_. B16-F10 melanoma cell line (ATCC) and mM1 pancreatic tumor cell line derived from KPC (LSL-Kras^G12D/+^;LSLTp53^R172H/+^;Pdx-1-Cre) model^74,36^ (kindly provided by Professor David Tuveson, CSHL) were cultured in DMEM medium with D-glucose, L-glutamine and pyruvate (Gibco, 41966-029), supplemented with 10% FBS (Sigma) and 1% Penicillin-streptomycin (Life Technologies). Murine non-tumour pancreatic fibroblasts (Pan Fib) and pancreatic cancer associated fibroblasts (Pan CAF) were derived from autochthonous KPC (LSL-Kras^G12D/+;^LSLTp53^R172H/+;^Pdx-1-Cre) tumors and kindly provided by Tobias Janowitz^48,74^. Briefly, CAFs were isolated from pancreatic tumour-bearing mice by mechanical separation of the tumor followed by digestion with an enzymatic cocktail consisting of 1 mg/mL collagenase A and collagenase D and 0.4 mg/Ml DNase I (all from Roche) in PBS at 37°C for 2–3 h with rotation at 600 rpm in a thermomixer compact (Eppendorf). 10 mM EDTA was then added to stop the enzymatic reaction. Normal fibroblasts (Fibs) were isolated from matched tissues of littermate controls using the same method.^48^ Pan CAF and Pan Fib were grown in complete RPMI medium: RPMI 1640 medium with L-glutamine and sodium bicarbonate (Sigma, R8758-500 mL) supplemented with 10% FBS, 1% Penicillin-streptomycin, 10 mM HEPES (Gibco, 15630-056) and 15 μM β-mercaptoethanol (Sigma, M7522-100 mL). Cells were split using trypsin. Pan CAF was split under differential trypsinization. Briefly, Pan CAF were split when reaching ~80–90% confluence; cells that detached quickly were collected and kept in culture for about 30 min, and cells that did not attach to the flask were removed and fresh medium was added. This method aims to eliminate tumor contaminant cells based on different properties of CAF to detach quickly from flasks and to attach quickly than tumor cells under trypsin treatment.

#### Murine tumors and tissue processing

To generate syngeneic tumors, female wild type C57BL/6JOla mice ~6–10 weeks old were subcutaneously injected in the flank with 50 μL of 1 × 10 ^6 mM1 cells resuspended in PBS and cultured as described above. For some experiments, mice were injected with the same volume of PBS as a control. Mice to be sacrificed at day 7 were occasionally injected on both sides of the flank, if material for imaging was necessary. Mice were sacrificed at days 7, 11, 13, 14 or 15 post-tumour injection. Tumors were measured during tumor development and measured/weighed at endpoint, with tumor volume calculated with the formula (π/6)*shortest length^2^*longest length. Animals were sacrificed before planned endpoint if tumors reached a diameter of 12 mm. If tumors started to ulcerate, mice were sacrificed, and tumors were not included in analysis. Tumors and blood were collected at endpoints for flow cytometry or imaging. Selective EP2 antagonist PF-04418948 (Cayman Chemical, 15016) and selective EP4 antagonist MF498 (Cayman Chemical, 15973) reconstituted in DMSO were administered together, intraperitoneally, in 150 μL topped up with PBS to reach a fixed dose of 10 mg/kg (calculated for average mouse weight of 20 g). Antagonists or vehicle control (PBS in DMSO) were administered for a total of 5 days from day 7 post-tumour injection till the day before endpoint. Mice were sacrificed at day 12 post-tumour injection, and tumors were collected for flow cytometry and imaging. For NK cell depletion experiments, anti NK1.1 mAb clone PK136 (BioXCell, BE0036) or respective isotype control, mouse IgG2a, k clone C1.18.4 (BioXCell, BE0085) were administered intraperitoneally every 3 to 4 days from day 2 post-tumour injection, at a fixed dose of 25 μg per injection and total of 4 doses.

Tumors were cut to smaller pieces and enzymatically digested for 1h15 at 600 rpm, 37°C in a shaker in 1 mL of RPMI medium with mg/mL of Collagenase A, 1 mg/mL Collagenase D and 80 μg/mL of DNase I (from Roche). Reaction was stopped with 10 mM EDTA in cold PBS, or with 10 mM EDTA in cold RPMI complete medium if cells were to be sorted and cultured afterward. Cells from digested tumors were smashed gently with a syringe plunger, passing through a 70 μM filter while washed with PBS 0.5% (m/v) BSA, if to be stained for flow cytometry, or with RPMI complete medium if to be sorted and cultured. Blood was resuspended in 5 mL of red blood cell (RBC) lysis buffer (prepared in house as: 150 mM ammonium chloride, 1mM potassium bicarbonate, 0.1 mM EDTA in distilled water or as 140 mM ammonium chloride, 17 mM Tris-base in distilled water, pH 7.2), incubating 5 min at room temperature and repeating this step if still bloody. Blood samples were washed with PBS 0.5% (m/v) BSA to proceed to flow cytometry.

Spontaneous genetic KPC (LSL-KrasG12D/+;LSLTp53R172H/+;Pdx-1-Cre) tumors were collected from mice aged approximately 32 weeks old (kindly provided by Tobias Janowitz^74^). Tumor samples were snap frozen in OCT (TissueTek) and 10μm sections were prepared for IF staining.

#### *Ex vivo* culture of iCAF in collagen/GelMa hydrogels

Day 12 or 13 mM1 tumors implanted in CAG.EGFP:αSMA-RFP mice were processed as described and iCAF sorted as: single live cells, CD45^−^ CD31^−^, GFP^+^, Podoplanin^+^, α-SMA-RFP^−^, CD34^+^ cells. Myofibroblastic (my)CAF were sorted as: single live cells, CD45^−^ CD31^−^, GFP^+^, α-SMA-RFP^+^ in BD Synergy sorters. Cells were cultured in a mix of 1:1 collagen/GelMa gels. Rat tail type I Collagen (Corning, 354236) was prepared accordingly to manufacturer instructions to a final concentration of 2 mg/mL. Briefly, total volume of gel to be prepared was calculated, and 10× phenol red PBS was added 1 to 10 to a separate tube on ice; then NaOH was added at 0.023 times the volume of collagen to be added in ml, followed by adding dH_2_O to account for all reagents to be mixed (total volume subtracted to NaOH, 10× PBS and collagen volumes). Lastly, collagen was added to the tube and mixed carefully, keeping it on ice till use. GelMa (Sigma, 900622-1G) was prepared accordingly to manufacturer instructions. Photoinitiator LAP (Sigma, 900889-5G) was first dissolved in PBS at 0.5% (m/v), followed by adding GelMa to make a final concentration of 8% (m/v), keeping it protected from light and incubating in an oven at 60°C for 2-3h, stirring occasionally, till completely dissolved. GelMa stock was aliquoted and stored at −20°C till use. To mix collagen and GelMa to make gels for cell culture, GelMa was thawed at 37°C while transferring collagen briefly to room temperature, protecting both from light. GelMa was added to collagen at a 1 to 1 ratio and mixed carefully, adding 200 μL in 48-well plates and letting collagen to polymerise for 20 min at 37°C, 5% CO2. To finish GelMa/collagen polymerisation, gels were subjected to short pulses of UV light (405 nm) till complete gel polymerisation was achieved. Gels were carefully washed twice with RPMI complete medium, keeping the plates in the incubator at 37°C, 5% CO2 with 300μL of RPMI complete medium till plating the cells. A minimum of 20,000 iCAF per well was cultured in collagen/GelMa between 3 and 4 days, observing the cell morphology and maintenance of iCAF phenotype (i.e., no α-SMA-RFP expression, confirmed under a fluorescence EVOS microscope, 10× magnification). Pre-activated NK cells (using 100 U/mL IL-2 for 48h) were added to iCAF wells at a ratio of NK:iCAF 1.5 to 1, incubating for 24h in RPMI complete medium supplemented with 100 U/mL IL-2. As a control, 10,000 Pan CAF were cultured in gels for 2 days followed by adding the same number of NK cells as to iCAF cultures. The following day, eBioscience™Cell Stimulation Cocktail was used to stimulate NK cells in the gels. After incubation, collagen/GelMa gels with NK cells and CAF were digested by adding 50 μL of 10mg/mL collagenase A and D directly into wells, incubating for 10 min or till complete gel digestion at 37°C, 5% CO2. Reaction was stopped with 10 mM EDTA, followed by washing the cells in cold RPMI complete medium twice. Cells were then stained for flow cytometry, analysing the degranulation and cytokine release capacity of NK cells.

#### Primary NK cells isolation and culture

Wild type C57BL/6JOla mice ~8–14 weeks of age were sacrificed, and spleens collected for primary NK cell isolation. Spleens were gently smashed with a syringe plunger while passing through a 70 μM strainer and washed with PBS. Cells were resuspended in RBC lysis buffer, incubating for 5 min at room temperature, and then resuspended in PBS. NK cells were isolated with the NK cell isolation kit (Miltenyi, 130-115-818) in LS columns (Miltenyi, 130-042-401) and a QuadroMACS or MidiMACS separator (Miltenyi), following manufacturer’s instructions. Negative fractions were collected and isolated NK cells were counted. Before being used in functional assays, NK cells were pre-activated with 300 U/mL IL-2 (Peptrotech, 212-012) in complete RPMI medium described for Pan CAF and Pan Fib, for 48h (0.5 × 10 ^6 cells/mL in 96 well U bottom plates at final volume of 200 μL).

#### Primary fibroblasts isolation and culture

Wild type C57BL/6JOla mice ~8–14 weeks of age were sacrificed, shaved, and skin from the flank was collected for primary fibro-blast isolation and culture. Skin was cut to smaller pieces and enzymatically digested in RPMI medium with 1 mg/mL of Collagenase A, 1 mg/mL Collagenase D and 80 μg/mL of DNase I (Roche), incubating for 1h15 at 600 rpm, 37°C in a shaker. Reaction was stopped with 10 mM EDTA in cold complete RPMI complete medium. Samples were smashed gently with a syringe plunger, passing through a 70 μM filter while washed with RPMI complete medium, twice. Cells were filtered again using a 50 μM strainer. 2 × 10 ^6 cells were plated in 24-well plates in 1 mL of RPMI complete medium, described for Pan CAF and Pan Fib. Cells in suspension were removed 30 min later, replacing it with fresh RPMI complete medium (differential adhesion method to obtain fibroblasts). Medium was replaced every two days for about 6–7 days, till cells with spindle shape morphology were visible under the microscope. Fibroblast purity was confirmed by flow cytometry. 1 × 10 ^4 fibroblasts were plated in 48-well plates for NK cell co-cultures, or 5 × 10 ^4–1 × 10 ^5 fibro-blasts were plated in 6-well plates for RNA extraction for RT-qPCR. Fibroblasts were stimulated with 20 ng/mL of recombinant murine IL-1α, TNF-α, IL-1α plus TNF-α, IFN-γ or TGF-β beta for 72h (Peprotech), refreshing medium 48h later. After 72h, fibroblasts were washed three times with RPMI complete medium, to minimise cytokine presence in further assays. Fibroblasts were lysed with 350 μL of RLT lysis buffer (Qiagen RNeasy Mini kit, 74106) and saved at −80°C for further RNA extraction, harvested for flow cytometry analysis, or kept for co-culture with pre-activated NK cells.

#### Generation of conditioned media

When Pan CAF, Pan Fib and mM1 reached ~80–90% confluence determined by observation under the microscope, they were split at a 1 to 10 dilution. The next day (confluence of ~40–50%), cell medium was replaced by fresh RPMI complete medium (Pan CAF and Pan Fib) or DMEM complete medium (mM1). The medium was collected the following day (confluence ~80–90%), centrifuged at 500 g for 6 min and the supernatant filtered with Steriflip Milipore Express, PLUS membrane (0.22 μM). When testing the role of COX-2 inhibitor NS398 (Cayman Chemical, CAY70590-5 mg), conditioned medium was generated as described above, but adding 4 μM of NS398 when refreshing the medium in the 24h before collection. All conditioned media generated was aliquoted and kept at −80°C for *in vitro* assays.

#### Killing assays

mM1 or B16-F10 were labeled with CellTrace™ CFSE (Invitrogen, C34554) and Pan CAF and Pan Fib cell lines with CellTrace™ Far Red (Invitrogen, C34564). For CellTrace™ CFSE labeling, cells were resuspended in 1 mL of RPMI 1640 medium with L-glutamine and sodium bicarbonate with 1% FBS and 5 μM CellTrace™ CFSE, incubating 5 min at room temperature, followed by washing twice with 11 mL of RPMI complete medium. For CellTrace™ Far Red cell labeling, manufacturing instructions were followed: cells were resuspended in 1 mL of PBS and 1 μM CellTrace™ Far Red and incubated for 15 min, washing in the same way described. Pre-activated NK cells and B16-F10 were co-cultured at a final volume of 100 μL in RPMI complete medium supplemented with 100 U/mL IL-2 (96-well U bottom plates), incubating for 24h at a ratio of NK:B16-F10 of 3:1 (2 × 10 ^4 B16-F10 cells). 2 × 10 ^4 mM1 cells or 2 × 10 ^4 mM1 plus 2 × 10 ^4 Pan CAF or Pan Fib were plated for 4h in 48-well plates. NK cells were added on top of cultures at a ratio of NK:mM1 2:1, incubating for 24h at 37°C, 5% CO_2_, in a final volume of 200 μL of RPMI complete medium, supplemented with 100 U/mL IL-2.

If testing the role of conditioned media, these were first diluted 1 to 1 in RPMI complete medium and supplemented with 100 U/mL IL-2. Conditioned media were added to the co-cultures during the killing assay or to NK cells in the 24h before the killing assay. In some experiments, solutions of 10–1000 ng/mL of mouse perlecan (Sigma, H4777), 0.5–2 μg/mL of mouse osteopontin (R&D Systems, 441-OP-050), 7–75 μg/mL mouse laminin (Invitrogen, 23017-015) were prepared in RPMI complete medium supplemented with 100 U/mL IL-2 and tested in killing assays. Controls with target cells (no NK cells) and incubating with the respective conditioned media were run in parallel to consider spontaneous cell death versus NK cell targeted killing. When testing Pan CAF conditioned media generated in the presence of NS398, controls with NS398 directly added to NK/tumor co-cultures were performed too. To test role of NKG2D and DNAM-1 in mM1 killing assays, NK cells were pre-incubated with 5 μg/mL of respective blocking antibodies and isotype controls (eBioscience,16-2261-81, clone 10E5, eBioscience, 16-4031-81, clone eB149/10H5, R&D Systems, MAB1547, clone 191004 and R&D Systems, MAB0061, 141945) in 100μL of RPMI complete medium for 30 min at room temperature; NK cells were then centrifuged and medium removed before proceeding to killing assays. In the case of the mM1 killing assay, cells were gently detached with Accutase® Cell Detachment Solution, confirming under the microscope that most cells were harvested for un-biased death quantification. The percentage of dead B16-F10, mM1, Pan CAF and Pan Fib and NK cell markers was analyzed by flow cytometry.

#### NK cell degranulation and cytokine release assays

2 × 10 ^4 Pan CAF and Pan Fib were plated overnight in 48 well plates, and the following day 4 × 10 ^4 pre-activated NK cells were added in RPMI complete medium supplemented with 100 U/mL IL-2. For testing the role of conditioned media on NK cell functions, these were prepared as described for killing assays and incubated with NK cells for 24h in 96 well U-bottom plates. For cytokine pre-treated fibroblasts/NK cell co-cultures, 3 × 10 ^4 pre-activated NK cells were added on top of fibroblasts previously cultured in 48-well plates, incubating for 24h in RPMI complete medium supplemented with 100 U/mL of IL-2. Cells were then stimulated with 10 ng/mL of phorbol-12-myristate-13-acetate or PMA (Sigma, P8139) and 0.5 μg/mL ionomycin (Abcam, ab120370) or with 1× eBioscience™ Cell Stimulation Cocktail (Invitrogen, 00-4970-93) in the presence of anti CD107a-PECy7 for 1h30 at 37 C 5% CO_2_. This was followed by 1× Brefeldin A (Biolegend, 420601) incubation for additional 4h at 37°C, 5% CO_2_. Cell stimulation and Brefeldin A treatment were performed simultaneously for 5h30 if CD107a staining was not required. Degranulation and cytokine release was assessed by flow cytometry.

#### Transwell migration assays

For NK cell migration assays, transwell 24-well plates (Costar, 3422) with 8 μM pore size were used. First, 600 μL of respective media were added in the wells, then 1 × 10 ^5 NK cells in 100 μL of RPMI complete medium were added in the inserts, settling first at 37°C, 5% CO_2_ for 10 min; the inserts were then transferred to the top of the respective wells. For NK migration assays toward distinct fibro-blast subtypes, these were generated and cultured to confluence as described. 24h before the migration assay, the fibroblast media was washed twice and replaced with 600 μL of RPMI complete medium; NK cells from *n* = 3 mice were counted and equally distributed on top of transwells as described, including controls of migration toward media. 2h30 later, cells on the bottom of wells (migrated) were collected and counted using a Neubauer chamber if migrating toward conditioned media only, or by flow cytometry staining if migrating toward fibroblasts on the bottom of wells (Precision Count Beads, Biolegend used for counting).

#### Flow cytometry

Murine cells were first resuspended in Fc block 1:300 (clone 2.4G2, BioXCell, CUS-HB-197) in PBS 0.5% (m/v) BSA, incubating on ice for 5 min. Cells were stained with fixable viability dye for 15 min at room temperature (Invitrogen, 65-0865-18 1:2000 or L34955 1:1000 dilution) followed by staining with extracellular antibodies in PBS 0.5% (m/v) BSA, incubating for 30 min at 4°C. If staining intracel-lularly, eBioscience™ Foxp3/Transcription Factor Staining Buffer Set (00-5523-00) was used. Cells were fixed in Fixation/Permeabilization buffer, incubating for 30 min at room temperature, followed by staining with intracellular antibodies in permeabilization buffer for 30 min at room temperature. Human PDAC samples were stained with a LIVE/DEAD™ Fixable Blue dye (Invitrogen, L23105A) for 15 min at room temperature and then Fc blocked (422302, Biolegend).

The following fluorochrome conjugated antibodies were used to stain mouse samples: Biolegend at 1:200 or 1:300 dilution, unless otherwise specified: anti CD107a (PE-Cy7, clone 1D4B), CD11b (APC-Cy7 or FITC, M1/70), CD11c (BV605, N418), NKG2A (APC or PE-Cy7 16A11), CD27 (PercPCy5.5, BV785 or BV421, LG.3A10), CD3 (AF488 or BV785, 17A2), CD31 (BV785, FITC, PercPCy5.5 or APC, clones 390 or MEC13.3), CD34 (BV421, MEC14.7), CD4 (PercPCy5.5, GM1.5), CD45 (BV785, APC or BV510, 30-F11), CD8a (BV785, 53-6.7), DNAM-1 (PE-Cy7 or PercPCy5.5, TX42.1), Granzyme B (PercPCy5.5, QA16A02), IFN-γ (BV421 or BV785, XMG1.2 or QA16A02), Ly49H (APC, 3D10), Ly6C (BV510, HK1.4), NK1.1 (PE or BV421, PK136 or XMG1.2), NKp46 (PE, 29A1.4), PD-1 (APC-Cy7 or BV605, 29F.1A12), PDGFR-α (PE-Cy7, APA5), Perforin (APC, 516009B), Podoplanin (APC-Cy7 or PE-Cy7, 8.1.1; 1:400 dilution), TNF-α (FITC, MP6-XT22), CD155 (PE-Cy7, TX56). The following BD antibodies were used at 1:200 dilution: anti CD49b (BV510, HMα2), NK1.1 (BUV395, PK136) and TIGIT (BV785, 1G9). The following Invitrogen antibodies were used at 1:200 or 1:300 dilution: anti CD3 (FITC, 17A2), CD34 (eFluor660, RAM34), FoxP3 (eFluor450, FJK-16s), Granzyme B (PE, NGZB), KI67 (PE-Cy, SolA15), MHC-II-I-A/I-E (eFluor450, M5/114.15.2), NKG2D (PE-Cy7 or PE, CX5), Perforin (APC, eBioOMAK-D), α-SMA (eFluor570, 1A4). R&D Systems antibodies anti RAE-1 (AF647, 199205) and anti CD155 (FITC, 829038) were used at 1:200 dilution. (The following anti-human Biolegend antibodies were used: anti CD45 (304038), CD3 (317344), NKp46 (331945), CD56 (362553), CD16 (302029); BD antibody anti CD11b (750065). Unstained, fluorescence minus one (FMO) and unstimulated NK cells were used as controls when appropriate. Samples were acquired in BD LSRFortessa machines or Cytek Aurora spectral flow cytometer.

#### Immunofluorescence imaging of murine tumors

Murine tumors were collected and snap frozen in O.C.T. compound (VWR, 361603E), keeping it at −80°C till sectioning. 12 μM tissue sections were generated in a Leica CM1520 cryostat and saved at −80°C again till staining. Tissue sections in slides were left to dry for 2h at room temperature and then fixed in 4% PFA for 10 min at room temperature. Samples were then washed 3 times in PBS, incubating 5 min per wash, and then blocked for 1h at room temperature in a humid chamber with block buffer: 3% (m/v) BSA, 2% (v/v) goat or donkey serum, depending on secondary antibodies to be used, 0.1% (v/v) Tween 20, 0.3 M glycine in PBS. Samples were then stained with unconjugated primary antibodies prepared in stain buffer: 3% (m/v) BSA, 2% (v/v) serum, 0.1% (v/v) Tween 20 in PBS, incubating overnight at 4°C in the humid chamber. The following day, sections were washed 3 times with 0.1% (v/v) Tween 20 in PBS and incubated for 1h15 in the humid chamber at room temperature with secondary antibodies (Invitrogen, used at 1 to 400 dilution) and conjugated primary antibodies, prepared in stain buffer. The following antibodies were used: goat anti-mouse PDGFR-α (R&D Systems, AF1062, 1:100), rat anti mouse perlecan (Invitrogen, MA1-06821, 1:300), Mouse/IgG2a, k anti mouse α-SMA (Invitrogen, 41-9760-82, 1:200), rat IgG2a, Rabbit IgG anti mouse Klrb1c/CD161c (NK1.1) (Cell Signaling, 39197, 1:200), hamster anti mouse podoplanin (Biolegend, 127404, 1:100), rat anti mouse CK19 (Merck, MABT913, 1:200) and rat anti mouse Ly6C (abcam, AB54223, 1:100). Samples were washed again 3 times with 0.1%(v/v) Tween 20 in PBS and stained with Fluoromount-G with DAPI mounting medium (Invitrogen, 004959-52). Slides were sealed with nail polish. Stained tumor sections were imaged at 20× magnification in an Olympus VS200 slidescanner microscope. Images were analyzed in Fij.

#### Enzyme-linked immunosorbent assays (ELISA)

ELISA kit KGE004B from R&D Systems was used to measure PGE2 levels in conditioned media following manufacturer’s instructions. Plates were read at 450 nm and corrected at 570 nm in a Tecan Absorbance microplate reader.

##### RNA extraction and one step RT-qPCR

RNA was extracted from cytokine-treated primary fibroblasts. Qiagen RNeasy Mini kit (Qiagen, 74106) was used following manufacturer’s instructions. RNA concentration was measured in a nanodrop, and 20 ng of RNA were used per reaction in one-step reverse transcription quantitative polymerase chain reaction (RT-qPCR). Preparation of samples for one-step RT-qPCR was performed using TaqMan™ RNA-to-CT™ 1-Step Kit (Applied Biosystems, 4392938) following manufacturer’s instructions. The following Taqman gene expression assays were used: *Ptgs2* (Mm00478374_m1), *Cxcl12* (Mm00445553_m1), *Cxcl10* (Mm00445235_m1), *Lif* (Mm00434761_m1), *Il6* (Mm00446190_m1) and *Acta2* (Mm00725412_s1). RT-qPCR reactions were run in a Viia 7 Life Technologies machine. Average Ct (threshold cycle) of duplicates was used to calculate relative gene expression. *Gapdh* was used as a reference gene and untreated fibroblasts were used as reference samples when calculating the relative gene expression applying 2 ^-ΔΔCT method.

##### Analysis of cell-to-cell distances in murine mM1 tumors

For quantification of NK cell distance to the closest CAF population, an ImageJ1 macro to be run in Fiji was developed in house. Briefly, the macro first resorts to the Stardist pre-trained neural network to segment the nuclei based on DAPI signal.^75^ A seeded watershed then is used to obtain the cell boundaries from the nuclei and an intensity mask computed on all channels.^76^ For each segmented region and each channel, the mean intensity is recorded and used to identify positive cells for the given channel using a global threshold. Next, combinations of markers are also considered. These events are mapped into the image and an euclidean distance transform enables to compute the proximity of the NK cells to the combination of markers. The average NK cell distance to each CAF population is returned by the macro. Counts of NK cells at a particular range of distances to CAF subtypes were obtained afterward by analysing the data in Excel. Brightness and contrast of microscopy images were adjusted across the whole scanned image for clear visualisation of the respective cells/regions. Areas with visible artifacts or in the periphery of whole scans were excluded from quantification (intratumoural regions quantified).

##### Analysis of publicly available datasets

Expression of PGE2 pathway related genes *PTGES2, PTGES, PTGES3* and *PTGES* in human pancreatic adenocarcinoma compared to control samples, and respective correlation with overall survival was assessed using Gepia2 RNA-sequencing expression data taken from the publicly available TCGA and GTEx projects.^51^ Publicly available mouse single-cell RNA sequencing data from KPC tumors at GEO: GSE129455.^11^ was analyzed using Partek™ Flow™ Explore Spatial Multiomics Data using Partek™ Flow™ software, v11.0.^77^ Only fibroblast-enriched samples were analyzed. iCAF, apCAF and myCAF clusters were identified on UMAP plots, based on markers described by the authors.^11^

#### Quantification And Statistical Analysis

Excel was used for data analysis in general, usually followed by GraphPad Prism v10 for plotting data and performing statistical analysis. FlowJo v10.6.2 was used to analyze flow cytometry data. Fluorescence microscopy images were visualised and analyzed with Fiji. Quantification of distance of NK cells to CAF was analyzed with an ImageJ1 macro run in Fiji and developed in house, as described. Mass spectrometry data was generated before,^48^ being re-analysed here for the purpose of this work. Data was plotted as mean ± SEM (standard error of the mean). To determine statistically significant differences, 2-tailed unpaired t-tests were performed when comparing 2 groups. One-way ANOVA with Tukey’s multiple comparison test was performed to compare several groups with each other. two-way ANOVA with Šídák’s multiple comparisons test was used to compare two groups at several time points or regarding different cell populations. Statistically significant differences in correlation analysis were determined with 2-tailed Pearson’s correlation coefficient. For all tests above, statistically significant differences were determined for *p*-values ≤0.05. For the TCGA and GTEx databases using Gepia2, ^51^ statistically significant differences of gene expression between control and tumor samples were determined with ANOVA for *p*-value ≤0.01. Statistical significance in Kaplan–Meier curves comparing overall survival of patients with pancreatic adenocarcinoma with high vs. low gene expression was determined with log -rank test for *p* ≤ 0.05. For the publicly available RNA-sequencing data, Partek^77^ was used to identify differentially expressed genes with ANOVA as FDR (False Discovery rate) <1e−8, narrowing down to fold change −2 to 2. Similar criteria was applied to identify differentially expressed genes related to NK cell function (Table S1).

## Supplementary Material

Supplementary data related to this article can be found online at https://doi.org/10.1016/j.celrep.2026.117527.

Doc S2

Supp Figs 1-12

Table S1

## In brief

Cancer-associated fibroblasts (CAFs) represent an abundant and heterogeneous component of pancreatic ductal adenocarcinoma (PDAC) but their interplay with natural killer (NK) cells is largely understudied. Rodrigues et al. show that CAFs, particularly myofibroblastic (my)CAF modulate NK anti-tumor capacity. This is mediated partially by prostaglandin E2, affecting granzyme B expression.

## Highlights

- NK cells are rare infiltrates in PDAC with a non-cytotoxic profile
- TGF-β driven myCAF are intrinsic suppressors of NK cell anti-tumor functions
- Fibroblasts and CAFs downmodulate NK cell activating receptors
- PGE2 antagonism partially rescues NK cell anti-tumor functions

## Resource Availability

### Lead contact

Requests for further information and resources should be directed to and will be fulfilled by the lead contact, Jacqueline D. Shields, jacqueline.shields@nottingham.ac.uk.

### Materials availability

This study did not generate new unique reagents.

### Data and code availability

- This paper analyzes existing, publicly available data, accessible at Gene Expression Omnibus (GEO) GSE129455 (GEO: GSE129455).^11^ Expression of PGE2 pathway related genes *PTGES2, PTGES, PTGES3*, and *PTGES* from the publicly available TCGA and GTEx datasets and respective correlation with overall survival was assessed using Gepia2.^51^
- Datasets reported in this paper can be shared by the lead contact upon request.
- All original code for imaging analysis is publicly available and has been deposited at: https://github.com/jboulanger/MultiplexTissueColocalization
- Any additional information required to reanalyze the data reported in this paper is available from the lead contact upon request.
