# Supplementary material for: Cancer-associated fibroblast subtypes differentially modulate natural killer cells in cancer
Source: Cell Rep. Author manuscript; Available in PMC 2026 Sep 8. (PMC7619464; doi:10.1016/j.celrep.2026.117527)
Supplement: Supplementary file 2 [file EMS218159-supplement-Supp_Figs_1_12.pdf]

Cell Reports, Volume *45*

Supplemental information

Cancer-associated fibroblast subtypes differentially modulate natural killer cells in cancer

Leonor Nunes Rodrigues, Hafsa Munir, Ibone Thate Arrazola, Matthias M. Gaida, Christoph Eckert, Jerome Boulanger, Ana C.F. Ferreira, Andrew N.J. McKenzie, and Jacqueline D. Shields

SUPPLEMENTAL FIGURES

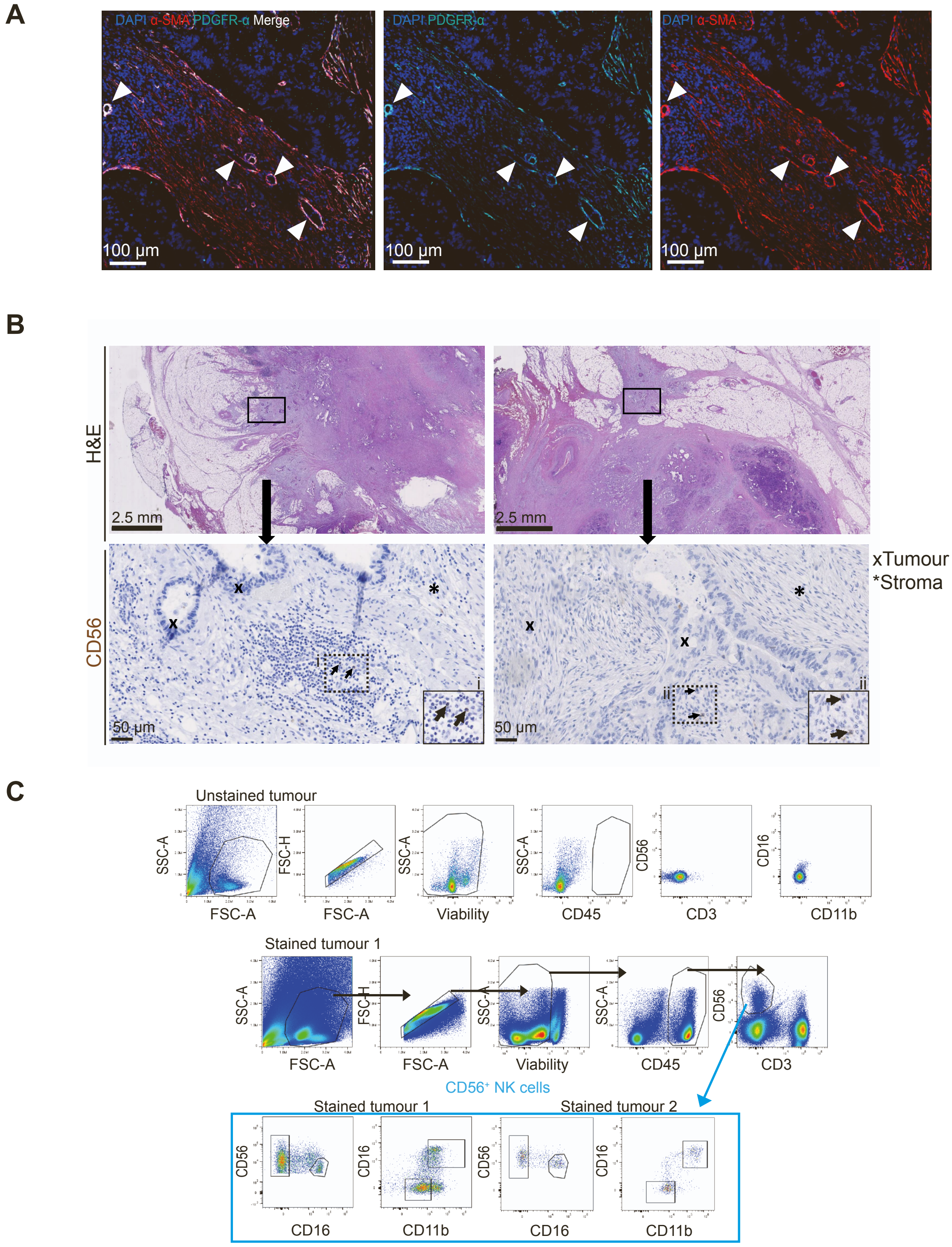

Figure S1 – NK cells are found in stromal regions in human PDAC. Associated to Figure 1.

A- Immunofluorescence staining of human TMA containing 34 and PDAC and 27 cancer-adjacent pancreatic tissues, exemplifying the staining of CAF and pericytes in blood vessels with very distinct shapes. Magnification 20X; DAPI (blue), α-SMA (red), PDGFR- α (cyan). White arrows indicate areas with blood vessel staining. Scale bar 100 μM.

B- H&E overview of 2 areas in one PDAC example (top panel). The squares mark areas at the invasive tumour margin; 20X magnification, n = 3 tumours analysed, scale bar 2.5mm. The lower panel shows similar regions at 400X of an immunohistochemical staining. Here, CD56^{+} NK cells (brown, identified

by arrows) are embedded in tumour stroma (*) near regions with tumour cells at the invasive margin (X), scale bar 50 μM.

C- Flow cytometry gating strategy for identifying and analysing NK cells in PDAC tumours.

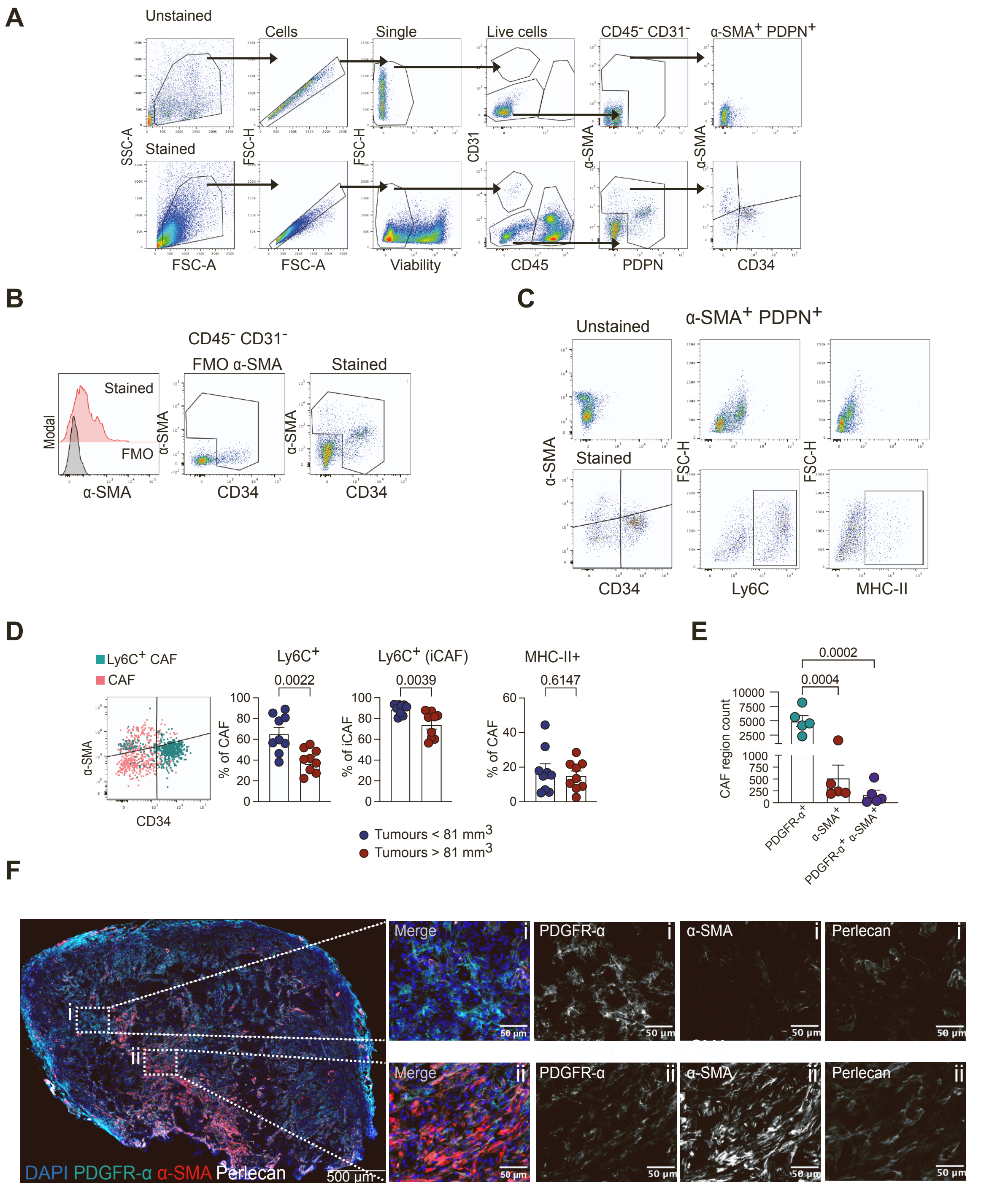

Figure S2 –mM1 tumour model retains CAF heterogeneity. Associated to Figure 2.

A- Flow cytometry gating strategy to analyse CAF in mM1 murine tumours, using an unstained tumour or FMO as a guide for gating.

B- Histogram and gating showing FMO control for α-SMA marker.

C- Gating of CD34, Ly6C and MHC-II on CAF, using unstained tumours for guiding the gating.

D- Ly6C as an iCAF marker: overlap of $Ly6C^+$ CAF with CAF, showing CD34 and α-SMA expression. Quantification of $Ly6C^+$ CAF based on average tumour volume and quantification of $Ly6C^+$ CAF as percentage of iCAF (α-SMA^- $CD34^+$). Quantification of MHC-II^+ CAF. 2 independent experiments, n=9 day 7 tumours and day 11 tumours, stratified based on average tumour volume of 81 mm^3.

E- Quantification of regions containing CAF subpopulations described in Figure 2 D; n= 5 tumours.

F- Microscopy image of day 11 mM1 tumour illustrating CAF architecture and ECM component perlecan, 20X magnification. DAPI (blue), PDGFR-α (cyan), α-SMA (red) and perlecan (white), scale bar 500 μM. Region of interest i highlights PDGFR-α$^+$ CAF lacking α-SMA expression. Region ii represents a region with α-SMA$^+$ CAF. Individual fluorescence channels displayed. Scale bar 50 μM.
Data shown as mean ± SEM and statistical significance determined with one way ANOVA with Tukey's multiple comparison test or two-tailed unpaired t-test for p-values ≤0.05. Relevant statistics shown.

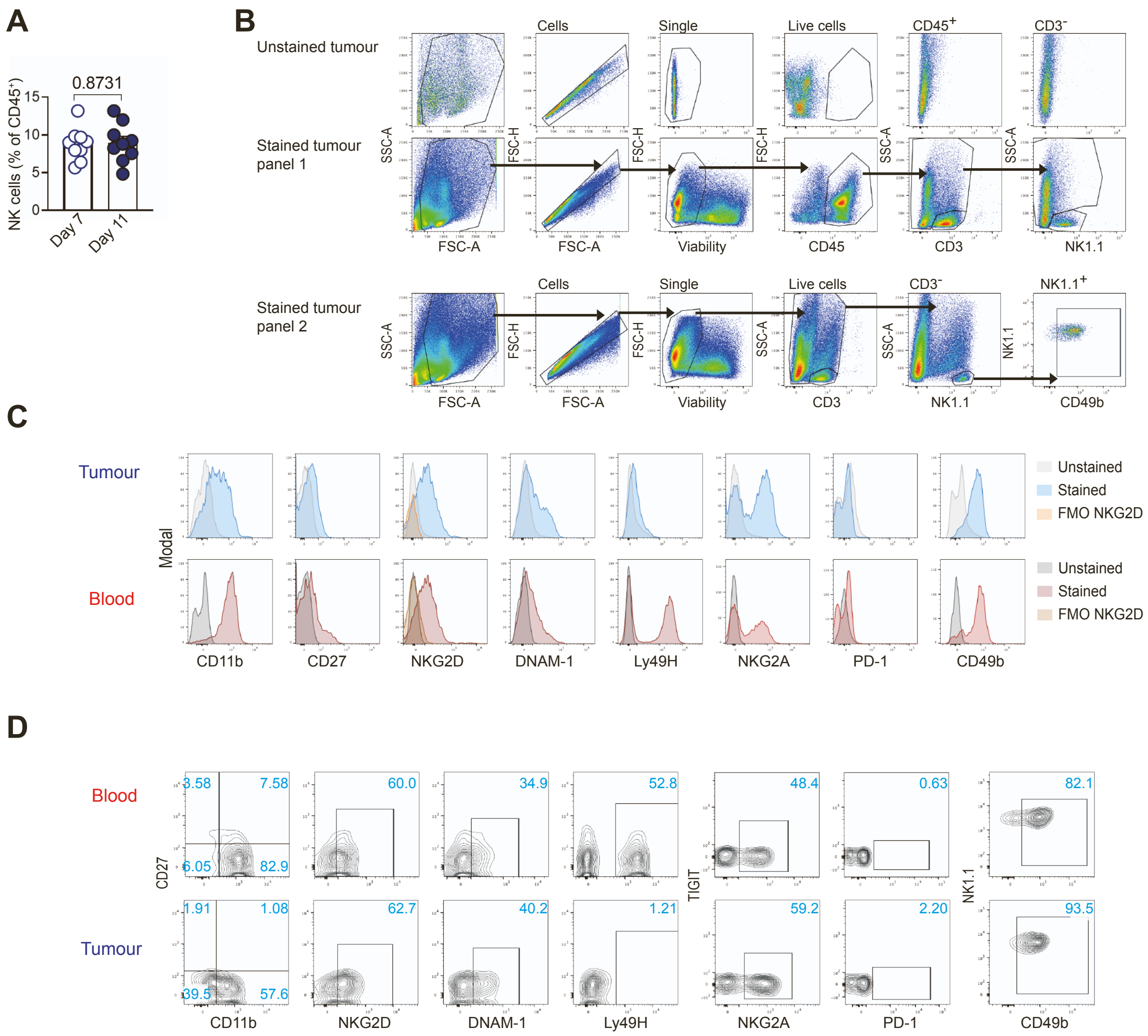

Figure S3– NK cells are immature intratumourally compared to blood counterparts. Associated to Figure 3.

A- NK cells as percentage of CD45$^+$ in mM1 tumours at days 7 and 11. Data from 2 independent experiments pooled, n = 9. Data shown as mean ± SEM and statistical significance determined with two-tailed unpaired t-test for p-values ≤0.05. Relevant statistics shown.

B- Gating strategy to identify NK cells within mM1 tumours in different flow cytometry panels, using an unstained tumour as a reference for gating.

C- Histograms of NK cell receptors comparing tumour with respective blood counterpart and unstained or FMO controls.

D- Gating of NK cell markers comparing blood with tumour.

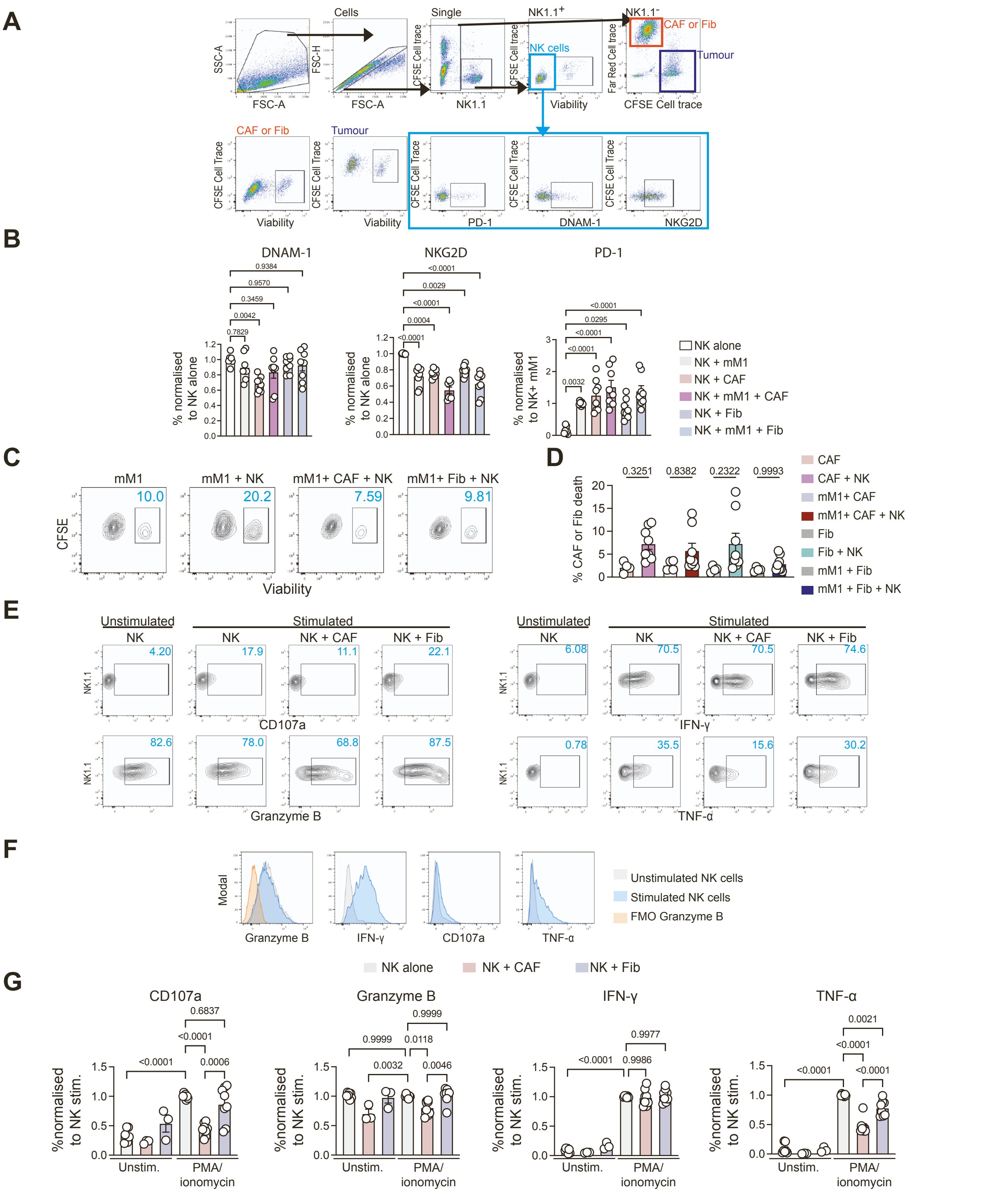

Figure S4– NK cells show decreased cytotoxicity and downmodulation of activating receptors when in contact with pancreatic CAFs *in vitro*. Associated to Figure 4.

A- Gating strategy to analyse tumour, CAF and Fib viability and NK cell markers in tri-culture killing assays.

B- Quantification of NK cell markers of cytotoxicity as percentage of NK cells normalised to "NK alone" (DNAM-1 and CD107a) and "NK + mM1" (PD-1) per experiment**;** n = 4 independent experiments; n=3 for controls "NK +CAF unstimulated" and "NK + Fib unstimulated"; technical duplicates for NK + CAF or Fib stimulated; technical duplicates for NK alone, except one experiment.

C- Flow cytometry gating illustrating viability of CFSE^{+} tumour cells when alone, in the presence of NK cells, NK cells plus CAF or Fib.

D- CAF or Fib death in killing assays; n = 4 independent experiments, technical duplicates for conditions with NK cells.

E- Flow cytometry gating for degranulation assay comparing the profile of unstimulated, stimulated NK cells and stimulated NK cells plus CAF or Fib.

F- Histograms representing unstimulated, stimulated and FMO for granzyme B of NK cell markers analysed.

G- Quantification of NK cell markers of cytotoxicity as percentage of NK cells normalised to “stimulated NK alone” per experiment (CD107a, granzyme B, IFN-γ and TNF-α). N=4 independent experiments; n=3 for controls “NK +CAF unstimulated” and “NK + Fib unstimulated”; technical duplicates for NK + CAF or Fib stimulated; technical duplicates for NK alone, except one experiment.

All data shown as mean ± SEM and statistical significance determined with one way ANOVA with Tukey’s multiple comparison test for p-values ≤0.05. Relevant statistics shown.

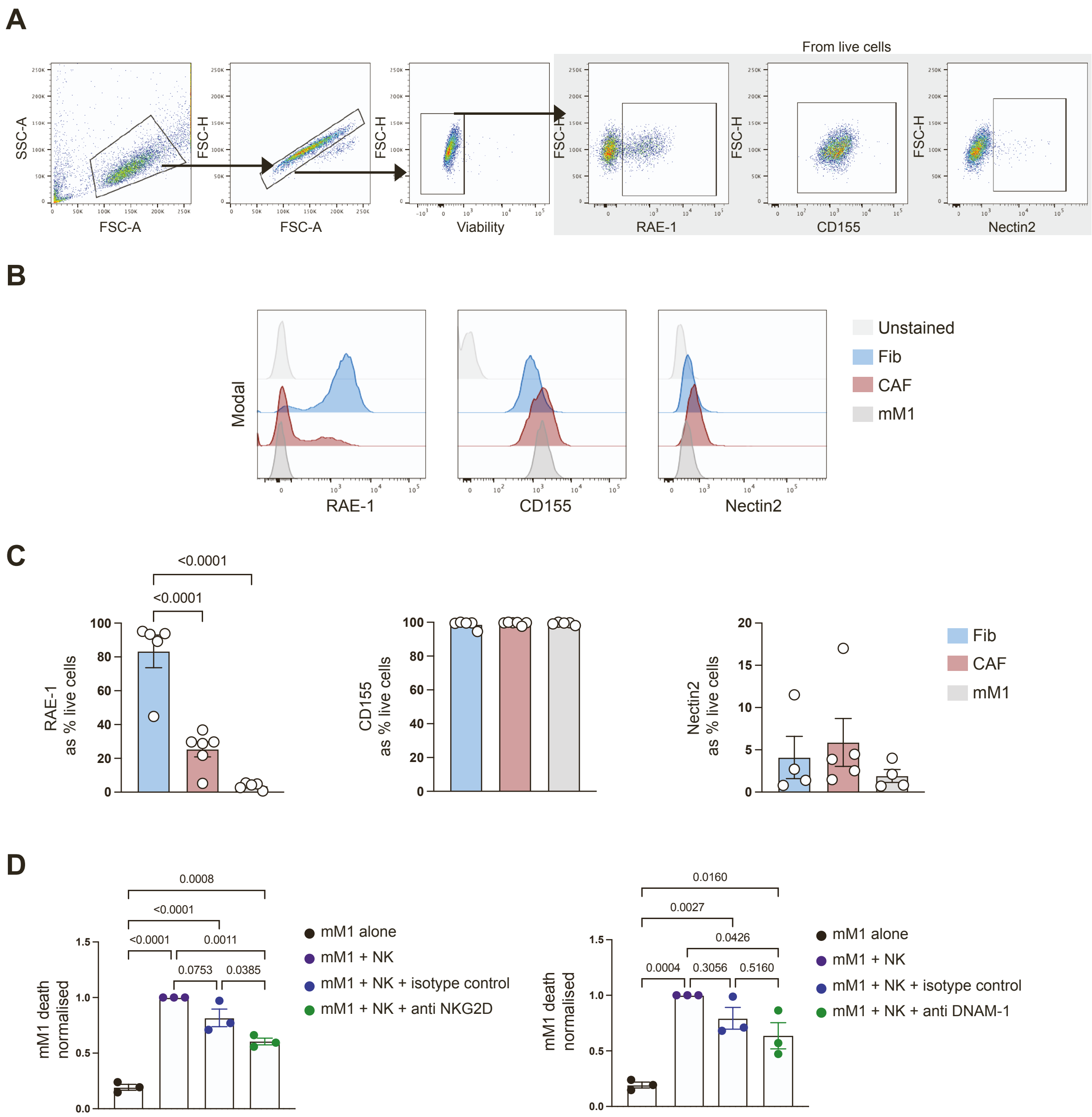

Figure S5– Fibs and CAFs express NK cell ligands which could mediate a decoy-like role protecting tumour cells from NK-mediated killing. Associated to Figure 4.

A- Flow cytometry gating strategy to analyse expression of RAE-1, CD155 and Nectin-2 in CAFs, Fibs and mM1.

B- Histograms showing the expression of markers described in A per cell type.

C- Quantification of expression of markers described in A as percentage of respective cell type; n = 4-6 (2 or 3 cell batches analysed in 2 or 3 independent experiments).

D- mM1 killing assays post treatment of NK cells with blocking antibodies to NKG2D and DNAM-1 and respective isotype controls; n=3 independent experiments.

All data shown as mean ± SEM and statistical significance determined with one-way ANOVA with Tukey's multiple comparison test for p-values ≤0.05. Relevant statistics shown.

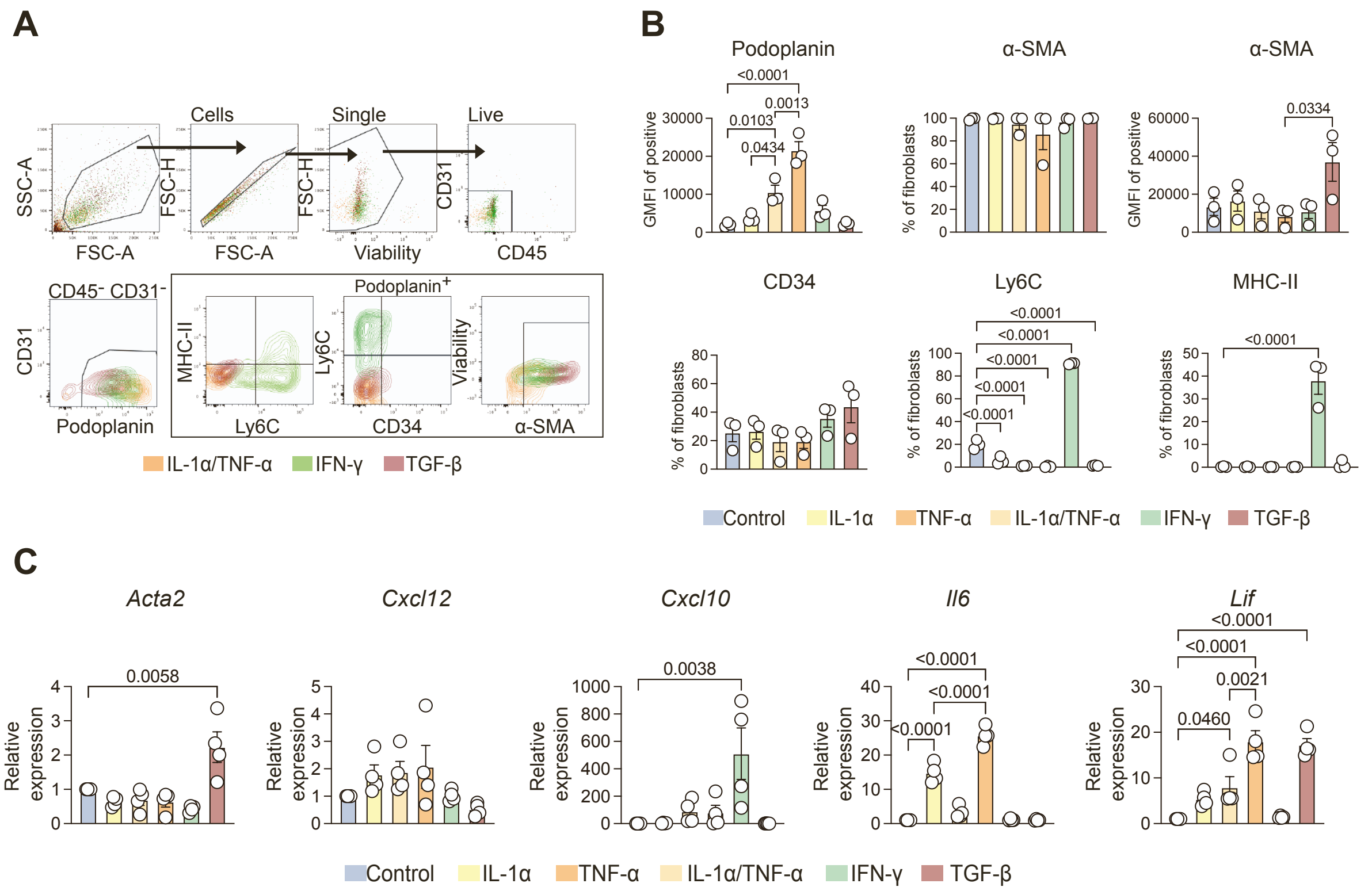

Figure S6– Different cytokines induce distinct functional profiles of fibroblasts. Associated to Figure 5.

A- Flow cytometry gating strategy of fibroblasts and distinct markers after cytokine treatments.

B- Flow cytometry quantification of fibroblast markers as GMFI of the positive population for podoplanin and α-SMA; quantification as percentage of fibroblasts for α-SMA, CD34, Ly6C and MHC-II. Fibroblasts were gated as CD45$^-$ CD31$^-$ podoplanin$^+$; n = 3 independent cultures performed.

C- Relative expression of fibroblast functional markers *Acta2*, *Cxcl12*, *Cxcl10*, *Il6* and *Lif* quantified with RT-qPCR as 2^-ΔΔCT using *Gapdh* as reference gene and fibroblasts as reference sample; n = 4 independent cultures.

All data shown as mean ± SEM and statistical significance determined with one-way ANOVA with Tukey's multiple comparison test for p-values ≤0.05. Relevant statistics shown.

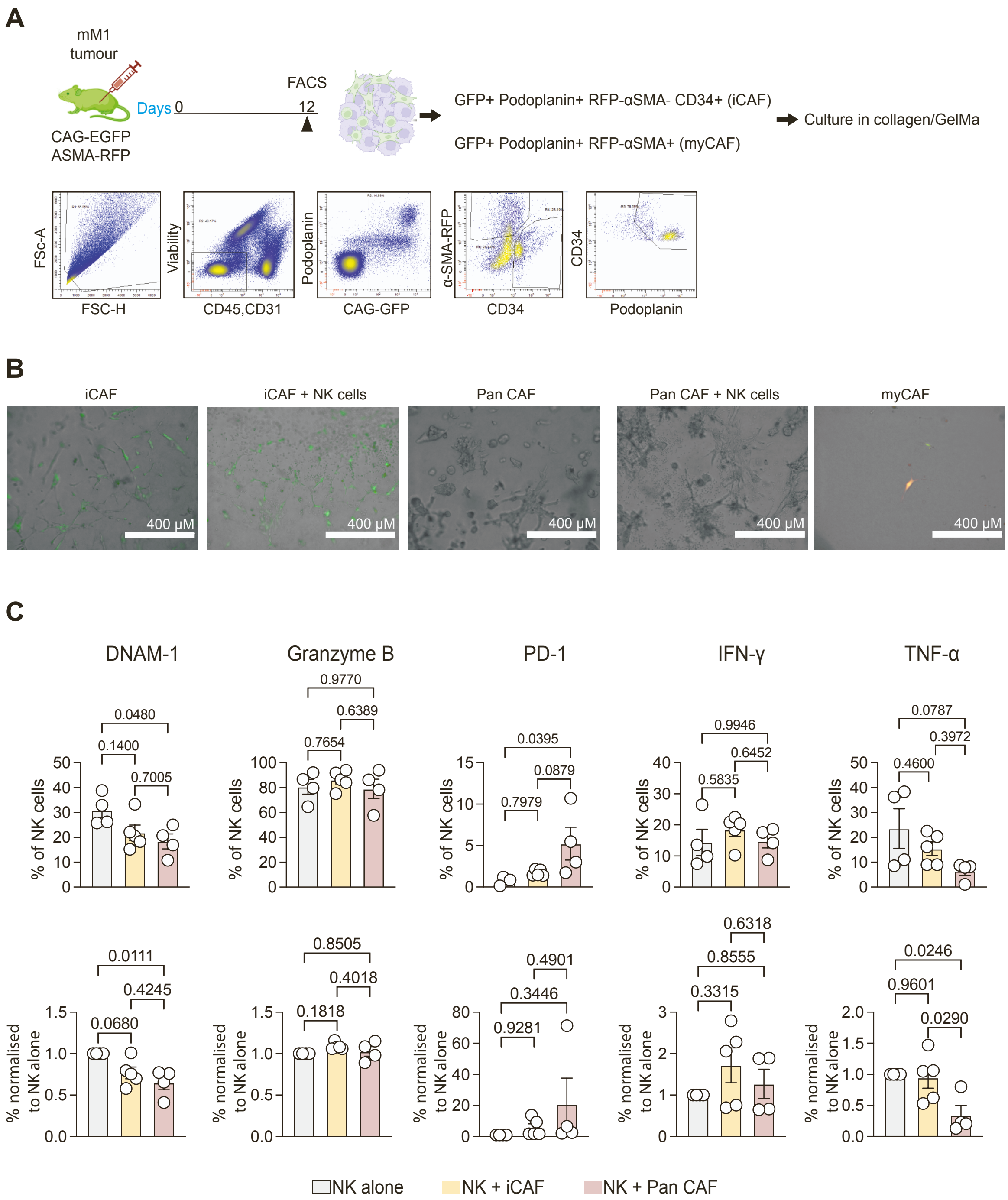

Figure S7– Tumour-derived iCAF do not suppress NK cell functions. Associated to Figure 5.

A- Illustration of mM1 tumour model on CAG-EGFP: α-SMA-RFP mice, cell sorting and culture (created with Biorender.com). Example of gating strategy used for sorting iCAF (single cells, live CD45$^-$CD31$^-$, GFP$^+$podoplanin$^+$, CD34$^+$ α-SMA$^-$ (podoplanin^{high}).

B- Images taken at 10X magnification of CAF/NK cell cultures in collagen/GelMa (phase, green-GFP, red- α-SMA). From left to right: iCAF, iCAF + NK cells, Pan CAF cell line, Pan CAF cell line + NK cells, myCAF. Scale bar 400 µM.

C- Flow cytometry quantification of NK cell functional markers DNAM-1, granzyme B, PD-1, IFN-γ and TNF-α as percentage of NK cells and normalised to NK alone per experiment; n = 4 independent cultures.

All data shown as mean ± SEM and statistical significance determined with one-way ANOVA with Tukey's multiple comparison test for p-values ≤0.05. Relevant statistics shown.

A

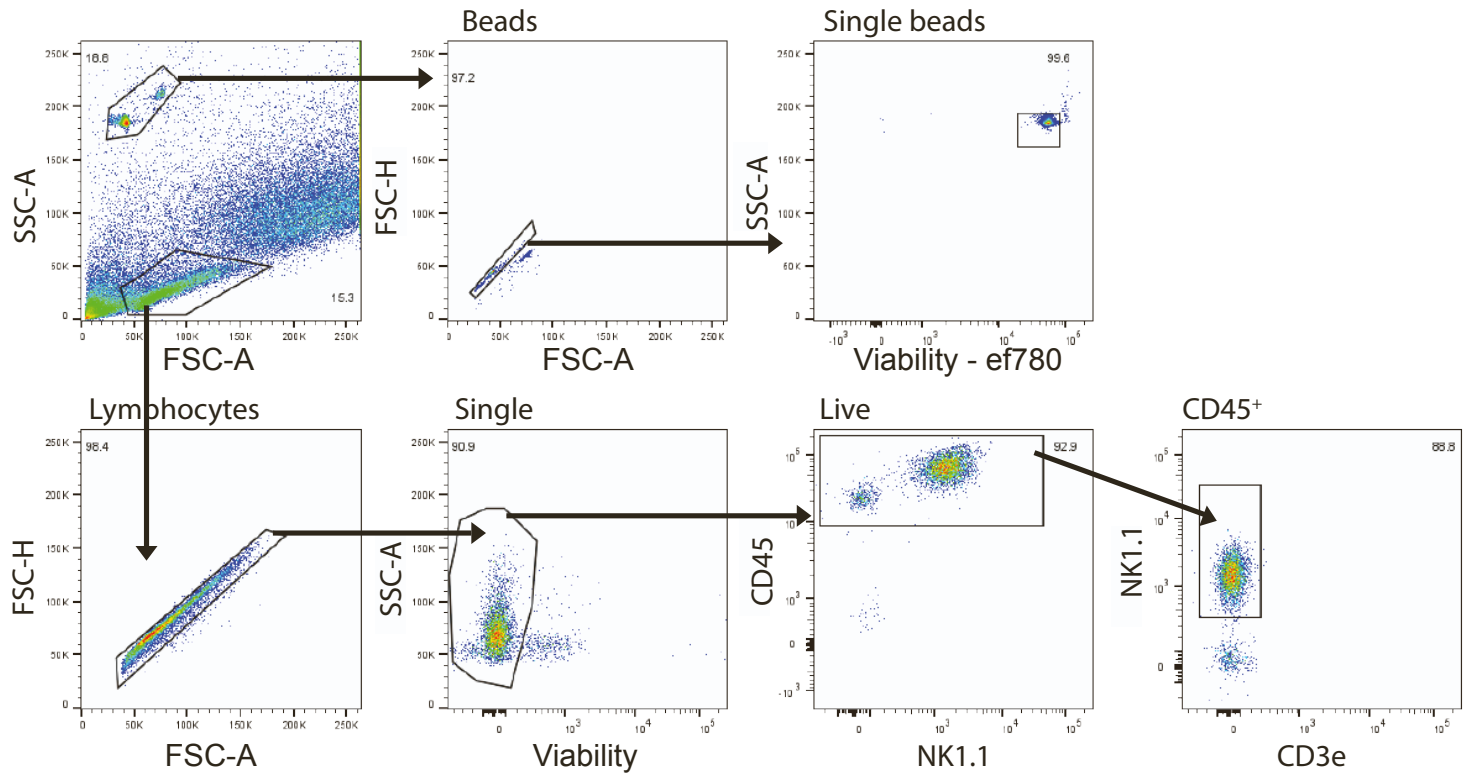

B

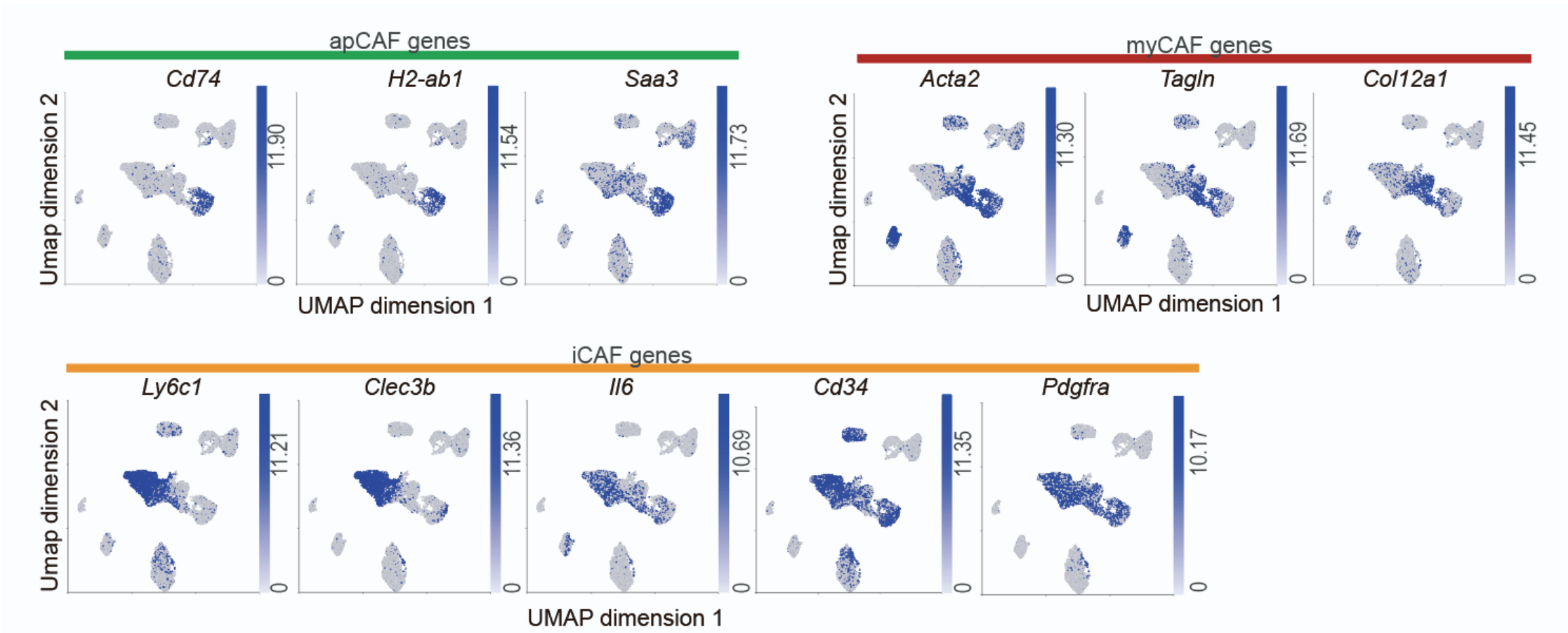

Figure S8– NK cells migrate towards fibroblast-derived factors. Associated to Figure 6.

A- Gating strategy used to identify counting beads and NK cells for subsequent counting in migration assays – associated to Figure 6 B.

B- Analysis of a publicly available single-cell RNA sequencing dataset consisting of a fibroblast-enriched fraction from KPC tumours [1]. UMAP plots showing the genetic expression of markers used to identify apCAFs, myCAFs and iCAFs.

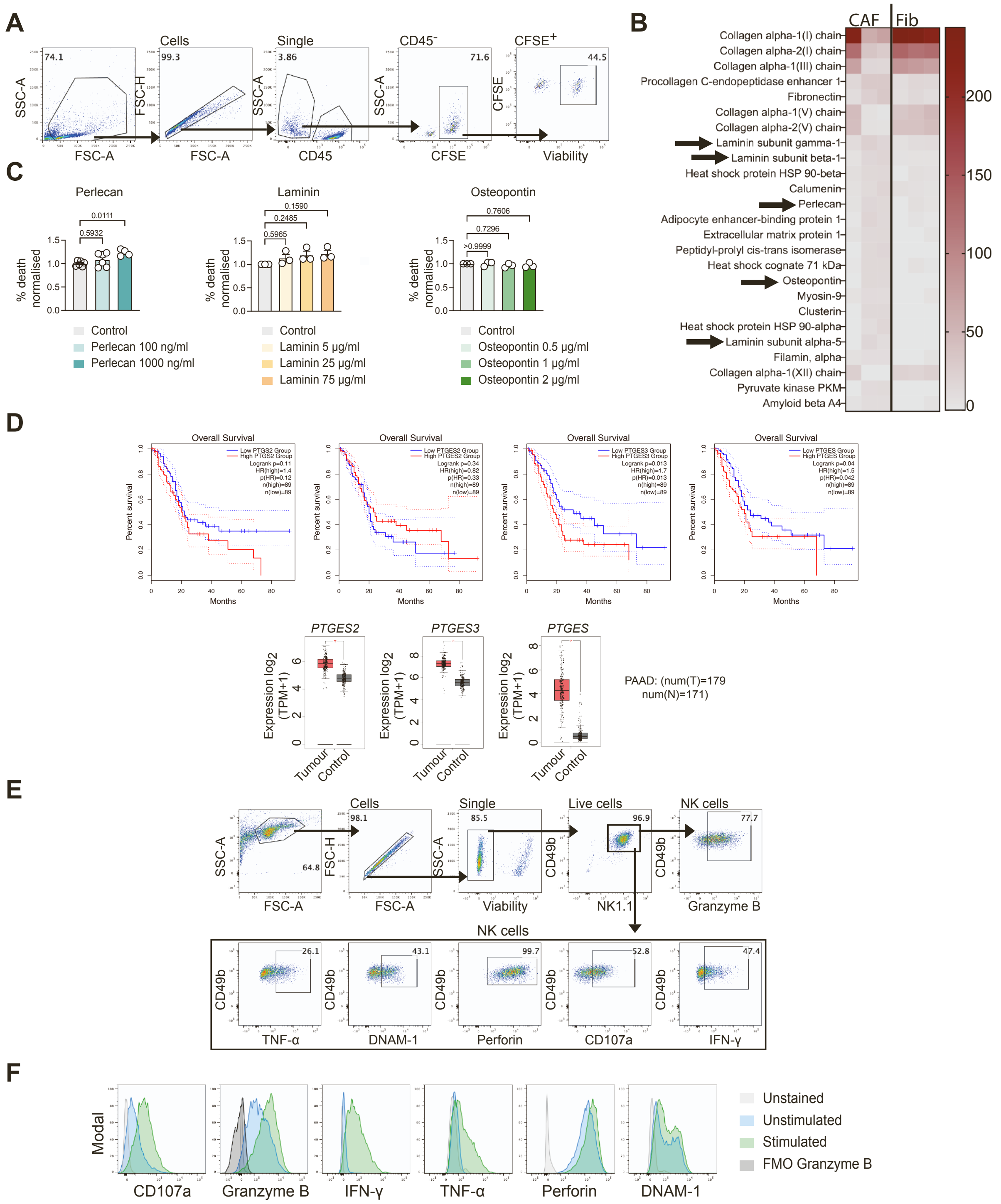

Figure S9– PGE2 is a CAF-derived soluble factor with therapeutic potential in pancreatic cancer. Associated to Figure 7.

A- Flow cytometry gating strategy for killing assays (tumour cells were CFSE stained, CD45⁻).

B- Heatmap showing reanalysis of mass spectrometry comparing the conditioned media of pancreatic CAF versus Fib cell lines [2]; 3 media batches tested per cell line. Arrows highlight targets of interest.

C- Quantification of tumour death in killing assay normalised to control media, to test NK cell killing capacity in the presence of perlecan, laminin and osteopontin. Perlecan: n=3 for 100 ng/ml and n=2 for 1000 ng/ml with technical duplicates; N=3 for all conditions in laminin and osteopontin. Killing assay performed with B16-F10 as target cells due to only soluble factors being tested. Data shown as mean ± SEM and statistical significance determined with one-way ANOVA with Tukey's multiple comparison test for p-values ≤0.05.

D- Analysis of publicly available datasets from TCGA and GTEx databases using Gepia2 [3]. Kaplan–Meier curves showing overall survival of patients with pancreatic adenocarcinoma high vs. low expression of *PTGS2*, *PTGES2*, *PTGES3* and *PTGES* [3]. Statistical significance determined with logrank test for $p \leq 0.05$. *PTGES2*, *PTGES3* and *PTGES* expression between control and patients with pancreatic adenocarcinoma [3]. Statistical differences determined with t-test for p-value ≤ 0.01.
E- Flow cytometry gating strategy for quantification of NK cell cytotoxicity and cytokine release after treatment with respective conditioned media.
F- Histograms of unstained, unstimulated, stimulated and FMO granzyme B for the respective NK cell markers analysed.

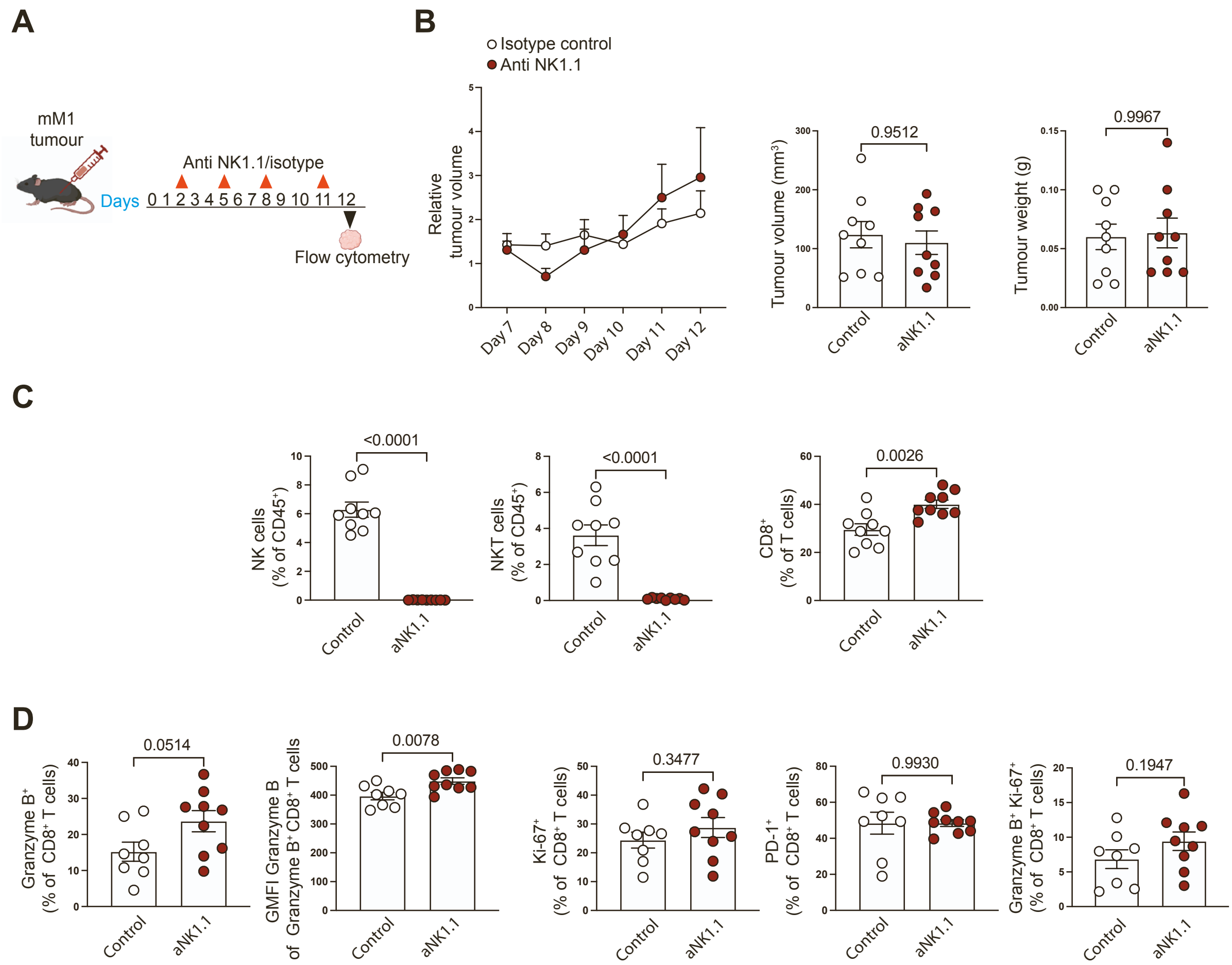

Figure S10– Tumour-associated NK cells suppress CD8^{+}T cell expression of granzyme B in mM1 tumours. Associated to Figure 7.

A- Illustration of mM1 tumour model under NK cell depletion (created with Biorender.com).

B- Relative tumour volumes (relative to first measurement), endpoint tumour volumes and weights.

C- Flow cytometry quantification of frequency of NK and NKT (as frequency of CD45^{+}) and CD8^{+} T cells (as frequency of T cells).

D- Analysis of CD8^{+} T cell profile: frequency of granzyme B^{+}, GMFI of granzyme B^{+}, frequency of Ki-67^{+}, PD-1^{+} and Ki-67^{+} granzyme B $^{+}$ (as % of CD8^{+} T cells).

2 experiments pooled, n=9 (isotype control), n=11 (anti NK1.1). All data shown as mean ± SEM and statistical significance determined two-way ANOVA with Šídák's multiple comparisons (for tumour volumes during tumour progression) or two-tailed unpaired t-test for p-values ≤0.05.

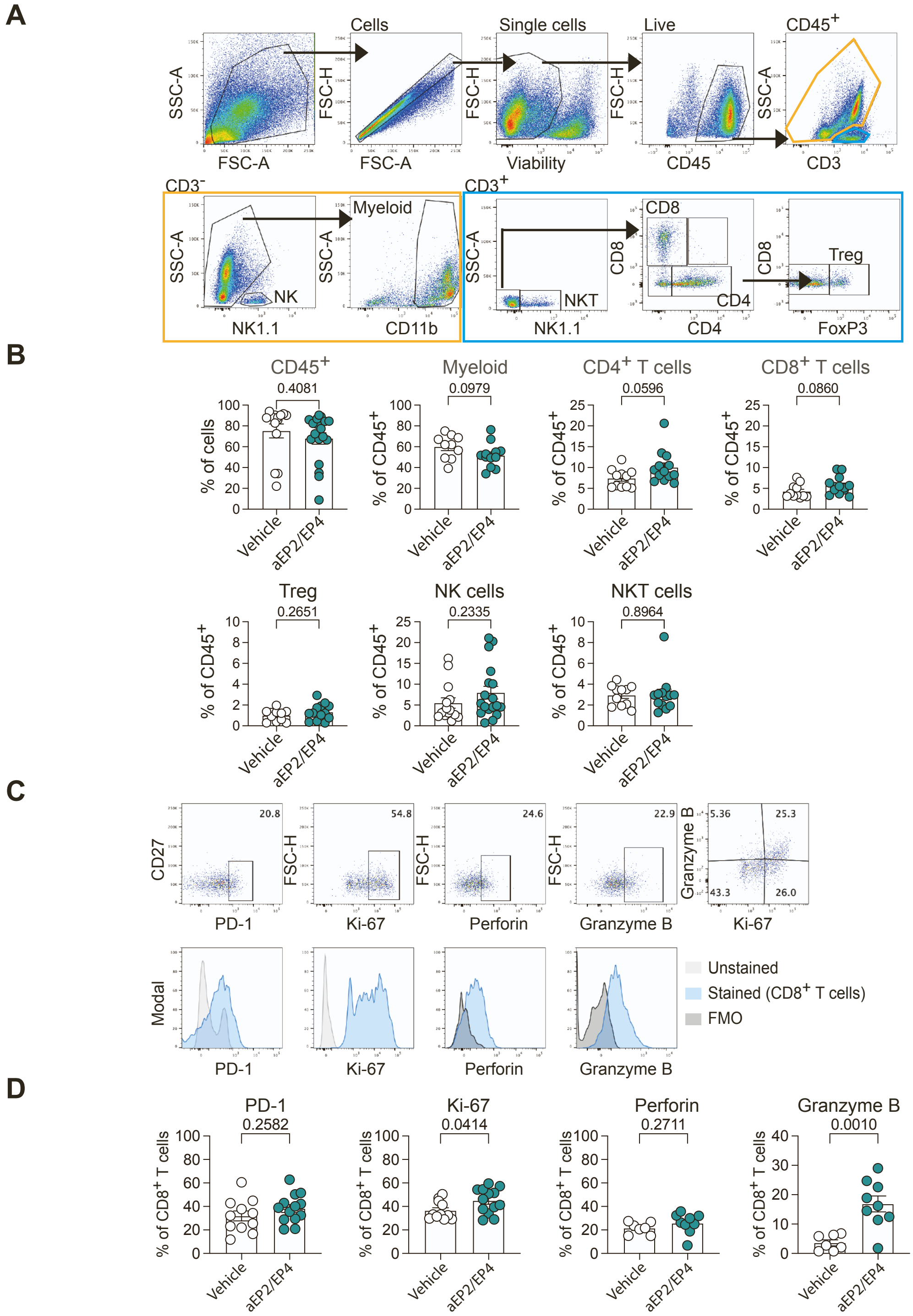

Figure S11– EP2/EP4 antagonism in mM1 tumours does not significantly change immune cell infiltration and profile. Associated to Figure 7.

A- Flow cytometry gating strategy showing how the distinct immune cell populations were identified.

B- Quantification of CD45$^+$ cells as percentage of live cells and of immune cell populations intratumourally as percentage of CD45+, comparing vehicle control with EP2/EP4 antagonism (aEP2/EP4). 4 experiments pooled for CD45$^+$, myeloid and NK cells: n= 14 and n=18 for vehicle and aEP2/EP4, respectively. 3 experiments pooled for T and NKT cells: n=11 and n=13 for vehicle and aEP2/EP4, respectively.

C- Flow cytometry gating strategy and controls with CD8$^+$ T cells as an example: PD-1, Ki-67, perforin and granzyme B.

D- Quantification of CD8+ T cell functional profile as gated in c, comparing vehicle with aEP2/EP4 treated animals. As percentage of CD8+ T cells: PD-1, Ki-67, perforin and granzyme B. 3 experiments pooled for PD-1 and Ki-67, n=10 and n=13 for vehicle and aEP2/EP4, respectively. 2 experiments pooled for granzyme B and perforin panel: n=7 (vehicle), n=9 (aEP2/EP4).
All data shown as mean ± SEM and statistical significance determined with two-tailed unpaired t-test for p-values ≤ 0.05.

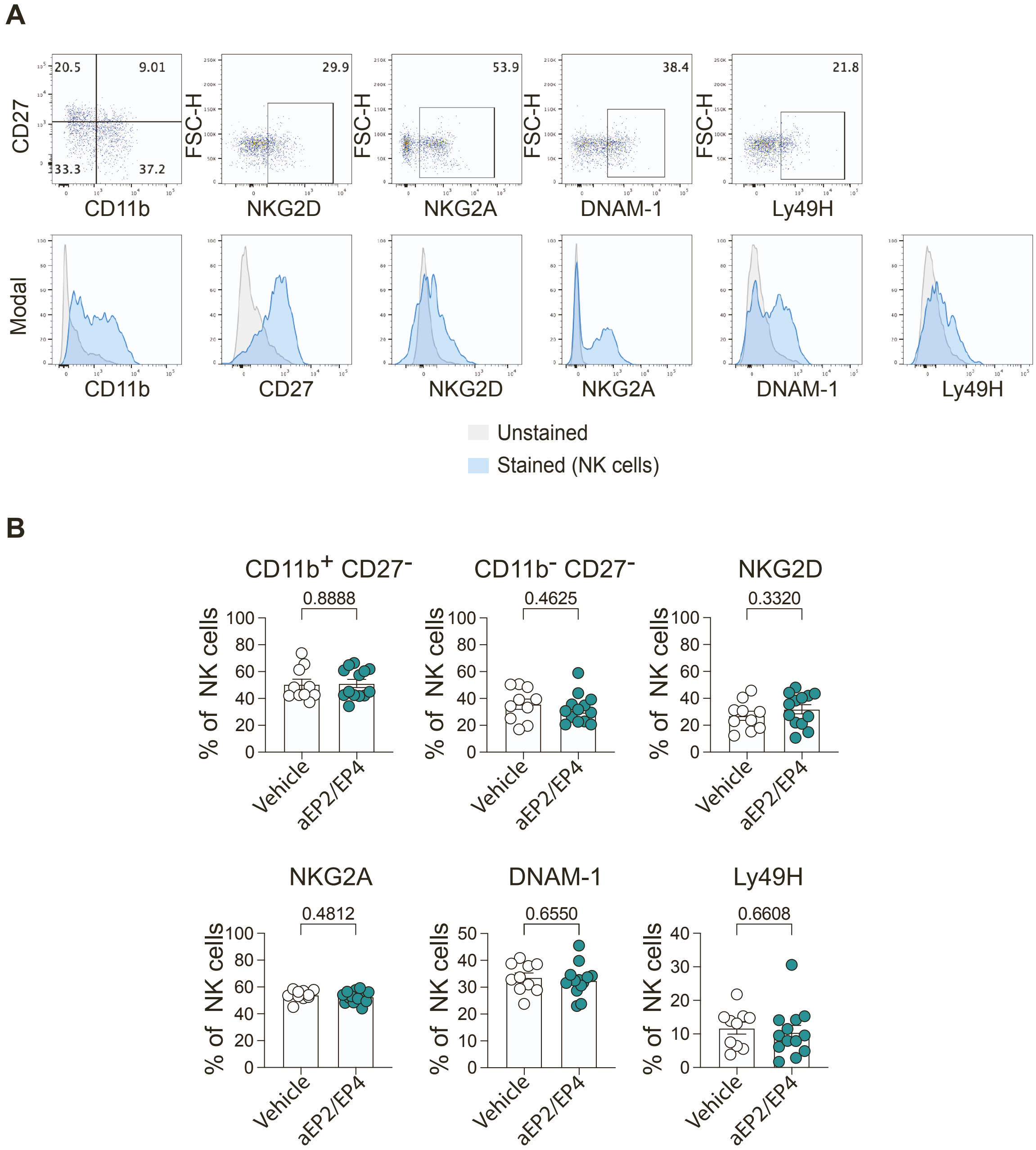

Figure S12– EP2/EP4 antagonism in mM1 tumours does not significantly change non-cytotoxic profile of tumour-associated NK cells. Associated to Figure 7.

A- Flow cytometry gating strategy and controls for NK cell markers CD11b/CD27, NKG2D, NKG2A, DNAM-1 and Ly49H, using unstained tumour as a guide for gating.

B- Quantification of NK cell markers as percentage of NK cells. 3 experiments pooled, n=10 and n=13 for vehicle and aEP2/EP4, respectively.

All data shown as mean ± SEM and statistical significance determined with two-tailed unpaired t-test for p-values ≤0.05.

SUPPLEMENTAL REFERENCES

1. Elyada, E., Bolisetty, M., Laise, P., Flynn, W.F., Courtois, E.T., Burkhart, R.A., Teinor, J.A., Belleau, P., Biffi, G., Lucito, M.S., et al. (2019). Cross-species single-cell analysis of pancreatic ductal adenocarcinoma reveals antigen-presenting cancer-associated fibroblasts. Cancer Discov. 9, 1102–1123. https://doi.org/10.1158/2159-8290.CD-19-0094,.

2. Munir, H., Jones, J.O., Janowitz, T., Hoffmann, M., Euler, M., Martins, C.P., Welsh, S.J., and Shields, J.D. (2021). Stromal-driven and Amyloid β-dependent induction of neutrophil extracellular traps modulates tumor growth. Nat. Commun. 12. https://doi.org/10.1038/S41467-021-20982-2,.

3. Tang, Z., Kang, B., Li, C., Chen, T., and Zhang, Z. (2019). GEPIA2: an enhanced web server for large-scale expression profiling and interactive analysis. Nucleic Acids Res. 47, W556–W560. https://doi.org/10.1093/NAR/GKZ430,.
